# The quest for epigenetic regulation underlying unisexual flower development in *Cucumis melo*

**DOI:** 10.1186/s13072-017-0132-6

**Published:** 2017-06-06

**Authors:** David Latrasse, Natalia Y. Rodriguez-Granados, Alaguraj Veluchamy, Kiruthiga Gayathri Mariappan, Claudia Bevilacqua, Nicolas Crapart, Celine Camps, Vivien Sommard, Cécile Raynaud, Catherine Dogimont, Adnane Boualem, Moussa Benhamed, Abdelhafid Bendahmane

**Affiliations:** 1 Institute of Plant Sciences Paris-Saclay (IPS2), CNRS, INRA, University Paris-Sud, University of Evry, University Paris-Diderot, Sorbonne Paris-Cite, University of Paris-Saclay, Batiment 630, 91405 Orsay, France; 2 Division of Biological and Environmental Sciences and Engineering, King Abdullah University of Science and Technology, Thuwal, 23955-6900 Kingdom of Saudi Arabia; 3 UMR 1313 Génétique Animale et Biologie Intégrative, Institut National de la Recherche Agronomique, 78350 Jouy-en-Josas, France; 4 UR 1052, Unité de Génétique et d’Amélioration des Fruits et Légumes, INRA, BP94, 84143 Montfavet, France

## Abstract

**Background:**

Melon (*Cucumis melo*) is an important vegetable crop from the *Cucurbitaceae* family and a reference model specie for sex determination, fruit ripening and vascular fluxes studies. Nevertheless, the nature and role of its epigenome in gene expression regulation and more specifically in sex determination remains largely unknown.

**Results:**

We have investigated genome wide H3K27me3 and H3K9ac histone modifications and gene expression dynamics, in five melon organs. H3K9ac and H3K27me3 were mainly distributed along gene-rich regions and constrained to gene bodies. H3K9ac was preferentially located at the TSS, whereas H3K27me3 distributed uniformly from TSS to TES. As observed in other species, H3K9ac and H3K27me3 correlated with high and low gene expression levels, respectively. Comparative analyses of unisexual flowers pointed out sex-specific epigenetic states of TFs involved in ethylene response and flower development. Chip-qPCR analysis of laser dissected carpel and stamina primordia, revealed sex-specific histone modification of MADS-box genes. Using sex transition mutants, we demonstrated that the female promoting gene, *CmACS11*, represses the expression of the male promoting gene *CmWIP1* via deposition of H3K27me3.

**Conclusions:**

Our findings reveal the organ-specific landscapes of H3K9ac and H3K27me3 in melon. Our results also provide evidence that the sex determination genes recruit histone modifiers to orchestrate unisexual flower development in monoecious species.

**Electronic supplementary material:**

The online version of this article (doi:10.1186/s13072-017-0132-6) contains supplementary material, which is available to authorized users.

## Background

Melon (*Cucumis melo*) belongs to the *Cucurbitaceae* family that includes about 800 species. Besides melon, the *Cucurbitaceae* family comprises several important vegetable crops, such as cucumber (*C. sativus*), watermelon (*Citrullus lanatus*), squash and pumpkin (*Cucurbita* spp.), and many neglected cultivated species that are major food crops in many developing countries. For decades, melon has been serving as a reference model organism for sex determination studies, and vascular fluxes [1, 2]. Melon also exhibits extreme genetic diversity for fruit traits, including fruit ripening, fruit shape, size, flesh color, texture, sweetness and aroma. Due to its relatively late entry into the genomic era, melon is a species with a barely explored epigenetic landscape. This limits the investigation of the cellular reprogramming of gene regulatory networks that drive development, growth and evolution of species of the *Cucurbitaceae* family.

Genome function, connectivity and structure rely on several epigenetic mechanisms that dynamically regulate the conformation of DNA, and thereby, its exposure to different regulators of gene transcription—e.g., transcription factors, non-coding RNAs, transposons [3–7]. At the chromatin level, DNA conformation can be modified by the addition or removal of chemical groups at specific N-terminal residues of histones [8, 9]. Some of these modifications are in charge of genome connectivity and heterochromatin stability, while others regulate gene expression or repression along the euchromatic regions. The distribution and abundance of these marks, at the gene and genome-wide scales, define an epigenetic landscape that directs cell differentiation and identity [10–13]. In the plant kingdom, genome-wide analyses of epigenetic landscapes have been performed in several species including *Arabidopsis* [14–17], maize [18–20], rice [21–23], common bean [24] and barley [25].

Histone methylation and acetylation are epigenetics marks that are usually associated with various biological processes ranging from transcriptional regulation to epigenetic silencing via heterochromatin assembly. While histone acetylation is commonly associated with transcription activation, histone methylation can either promote or inhibit gene transcription [5, 11, 18, 26–29]. Histone H3 lysine 9 acetylation (H3K9ac) is an epigenetic mark commonly found in active promoters [5, 20, 30, 31]. Genome-wide studies in *Arabidopsis* have found around 5200 non-Transposable Element (TE)-related genes that are targeted and regulated by H3K9ac [31]. In contrast, H3 lysine 27 trimethylation (H3K27me3) is commonly associated with gene repression, thereby contributing to the mitotic heritability of PRC2 (Polycomb repressive complex)-mediated silencing of numerous developmental and homeotic genes [29, 32, 33]. In the plant kingdom, the study of these epigenetic marks has elucidated the epigenetic component regulating plant growth and adaptation to their harsh environment [34–44].

The majority of angiosperms are hermaphrodite producing exclusively bisexual flowers. Sex determination is a developmental evolutionary process that leads to unisexual flowers. Monoecious species, such as melon, exhibit male and female flowers on the same plant. Dioecious species have separate male and female individuals [45, 46].

In melon, floral primordia are initially bisexual with sex determination occurring by the selective developmental arrest of either the stamen or the carpel primordia, resulting in unisexual flowers [47, 48]. This sexual organ arrest is genetically governed by the interplay of alleles of the *andromonoecious* (*M*), *androecious* gene (*A*) and *gynoecious* (*G*) genes [47, 49–51]. The cloning and characterization of *M*, *G* and *A* genes have shown that the *gynoecious* (*G*) gene encodes a zinc finger transcription factor, *CmWIP1* [47], the andromonoecious (*M*) gene and the androecious gene (*A*) encode ethylene biosynthesis enzymes: CmACS-7 [49] and CmACS11 [51], respectively. Genetic analysis revealed a mechanistic model in which expression of the carpel inhibitor, *CmWIP1*, is dependent on non-expression of *CmACS11* and expression of the stamina inhibitor, *CmACS*-*7*, is dependent on non-expression of *CmWIP1*. In monoecious plants, male flowers result from non-expression of *CmACS11* that permits *CmWIP1* expression. Female flowers develop on branches expressing *CmACS11*, which represses the expression of *CmWIP1*, and thus, releasing the expression of *CmACS7* inhibiting stamina development [51–53].

Now that the identity of the androecy, monoecy and gynoecy sex genes are revealed and genetic pathway controlling sex determination in *Cucurbitaceae* discovered, among the next challenges is to decipher how the sex determination signals are perceived and how the information is translated to cause organ-specific abortion at the flower and the plant level. Several studies have reported a wide-range of epigenetic processes and environmental cues that determine sex in animals [54–57]. In plants, it is well known that sex ratio and determination can be influenced by different environmental factors such as day length and light intensity [58], water restriction [59] and some plant hormones such as ethylene [49–51, 60–63], auxin [64] and gibberellins [65–67]. This multifactorial regulation of sex determination is still poorly characterized from the epigenetic point of view, where limited literature have reported the association for instance of small RNA (sRNAs) transcription and DNA hypomethylation with sex determination [54, 68–71].

In melon, the epigenetic control of sex determination remains poorly characterized and is mainly attributed to a transposon-induced DNA methylation that leads to gynoecy [47]. To gain new information on the epigenetic control of sex determination, we have examined the genome-wide landscape of H3K27me3 and H3K9ac histone modifications and gene expression dynamics, in five melon organs, focusing our analysis on unisexual flowers. The combination of epigenomic and gene ontology analysis pointed out sex-specific epigenetic states of TFs involved in ethylene response and sexual organs development. Furthermore, using sex transition mutants, we demonstrated that the female promoting gene represses the expression of the male promoting genes via deposition of H3K27me3.

## Results

### Genome-wide landscape of H3K9ac and H3K27me3 in melon

**Fig. 1:**
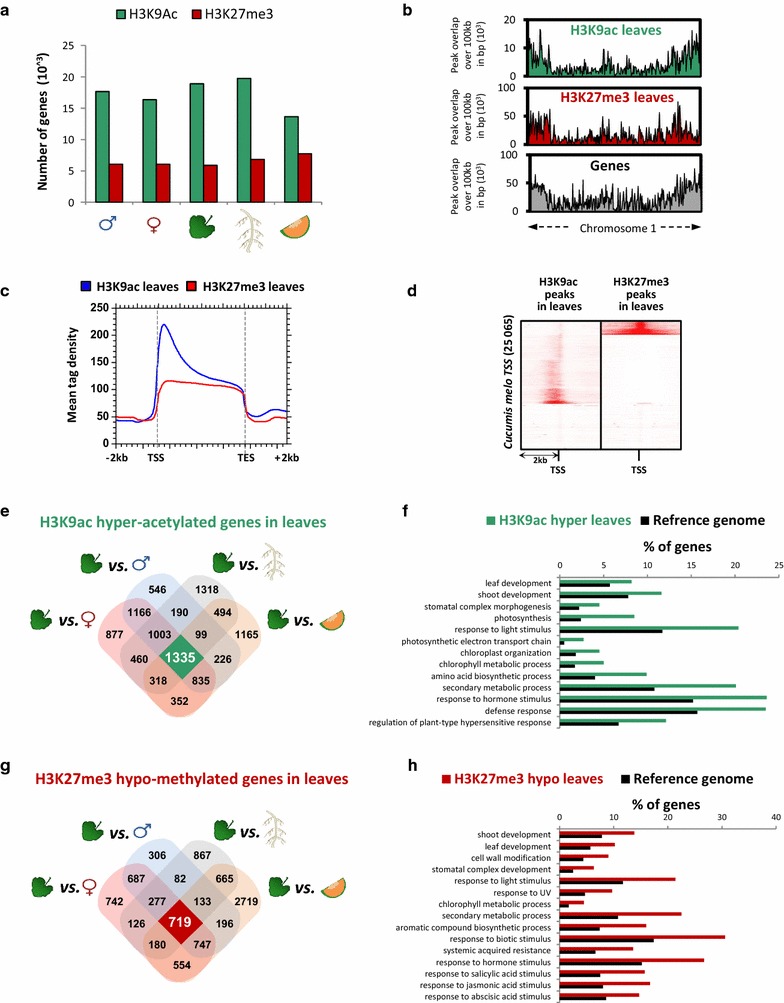
Genome-wide distribution and tissue-specificity of H3K9ac and H3K27me3 in melon. **a** H3K27me3 and H3K9ac target genes. Number of genes presenting H3K9ac (*green bar*) and H3K27me3 (*red bar*) peaks were quantified and plotted for the five different tissues. **b** H3K27me3 and H3K9ac distribution at the chromosome level. Distribution of H3K9ac (*green*, *upper panel*), H3K27me3 (*red*, *medium panel*) in leaves and annotated genes (*gray*, *bottom panel*) are plotted along the chromosome 1. **c** Average tag density profile of H3K27me3 and H3K9ac along the gene body. ChIP-Seq densities of equal bins were plotted along the gene body and 2-kb region flanking the TSS or the TES. **d** Correlation between the genome-wide distribution of H3K27me3 and H3K9ac. Heat map representing the tag density distribution of H3K27me3 and H3K9ac across all genes and a 2 kb flank. **e** Leaf-specific H3K9 hyper-acetylated genes. Paired comparisons of leaves vs. all the organs are shown as a Venn diagram, leaf-specific hyperacetylated genes correspond to the central overlap (highlighted in *green*). **f** GO terms of biological processes enriched for leaf-specific hyperacetylated genes. **g** Leaf-specific H3K27 hypo trimethylated genes. Paired comparisons of leaves versus all the tissues are shown as a Venn diagram, leave-specific hypo methylated genes correspond to the central overlap (highlighted in *red*). **h** GO terms of biological processes enriched for leave-specific hypo trimethylated genes. *p* values for each enriched class are presented in Additional file 9: Table VIII

Genome-wide studies in *Arabidopsis* [14–17], and other plant species such as maize [19, 20], rice [21–23], and barley [25], have evidenced the important role of H3K9ac and H3K27me3 in gene activation and repression, respectively. The roles of these histone modifications in melon development remain unknown and represent a limit to fully understand how thousands of bioprocesses are regulated. To determine the genomic landscape and the organ specificity of those marks, we performed ChIP-seq analyses using H3K27me3 and H3K9ac antibodies on different organs, namely fruits, leaves, roots, female and male flowers. Two replicates per tissue were processed and present a correlation coefficient of at least 0.99 (Additional file 1: Figures S1 and S2). A minimum of 14 millions of mapped reads were obtained in the replicates implemented (Additional file 2: Table I). The MACS2 algorithm, which is designed to detect typical histone peaks [72], was used to determine loci that are significantly enriched with H3K9ac or with H3K27me3 (Additional file 1: Figures S3 and S4). We identified 18,424 H3K9ac marked genes in leaves, 18,024 in roots, 13,655 in fruits, 17,660 in male flowers and 16,348 in female flowers. On the other hand, we identified 6486 H3K27me3 marked genes in leaves, 7741 in fruits, 6823 in roots, 6055 in male and 6054 in female flowers (Fig. 1a; Additional file 3: Table II).

Next we analyzed the distribution of those two marks at the chromosome and gene levels. For the sake of simplicity, we will describe mainly results obtained in leaves, and results obtained in the other organs are shown in the additional file. The chromosomal distributions of annotated genes, H3K9ac and H3K27me3 and in each organ on the 12 chromosomes, are illustrated in Additional file 1: Figure S3. At the chromosomal scale we observed an enrichment of both marks in gene-rich regions, which are localized at chromosome arms, compare to centromeric and pericentromeric regions, where the majority of transposable and retro transposable elements are located [73], consistent with the role of these histone marks in the control of gene expression (Fig. 1b; Additional file 1: Figures S5 and S6). In order to detail the H3K27me3 and H3K9ac distributions at the gene level, the peaks obtained in each tissue for both modifications were analyzed and averaged within a 4000-bp region, flanking the TSS and TES of all the annotated genes. We found that the peak length of H3K9ac ranged from 200 bp to 700 bp (Additional file 1: Figure S7), located preferentially at the TSS regions, which correspond to the first nucleosomes of the genes (Fig. 1c; Additional file 1: Figure S8). In contrast, H3K27me3 peaks presented an averaged length that ranged from 2500 to 2700 bp, which covered the entire gene body (Fig. 1c; Additional file 1: Figure S7). Those patterns were observed in all the organs (Additional file 1: Figures S7 and S8) and were consistent with previous studies on different plant species, highlighting conserved aspects of the epigenetic system in the plant kingdom. Integration of H3K9ac and H3K27me3 datasets showed a clear anti-correlation between those two marks (Fig. 1d). Altogether, these results showed that in melon, as in other plant species, H3K27me3 and H3K9ac are not enriched in intergenic regions but distributed along the gene body, supporting the role of these two marks in gene regulation in an exclusive manner.

### Identification of organ-specific H3K27me3 and H3K9ac landscapes

We next aimed to determine the organ-specific profile of both H3K27me3 and H3K9ac marks, and to determine whether these chromatin modifications contribute to the establishment of organ-specific gene expression programs. To this end, first, for each organ, we determined the genes that are marked either by H3K9ac or by H3K27me3 or by both. Secondly, we performed a comparison between all the possible paired combinations of the five different tissues using DiffReps, a highly sensitive program for the detection of differential sites from ChIP-seq data [74]. Third, a Venn diagram was generated to identify genes that were over-represented in the chosen organ regardless of which organ it was compared to and thus to fish out genes that are specifically marked in this organ (Fig. 1e, g; Additional file 1: Figures S9–S12; Additional file 4: Table III). To determine the biological function of these genes, we performed a gene ontology analysis (GO) using Plant MetGenMAP [75]. Organ-specific hyperacetylated and hypomethylated genes, displayed biological processes terms consistent with metabolic needs and physiological aspects of their corresponding organ, providing evidence for the robustness of our data (Fig. 1f, h; Additional file 1: Figures S9–S12). For example in leaves, we found 1335 specifically hyperacetylated genes involved in photosynthesis, leaf development, stomatal morphogenesis (Fig. 1f). Consistent results were also obtained for fruits, roots, female and male flowers (Additional file 1: Figures S9–S12).

These results suggest that both H3K9ac and H3K27me3 display an organ-specific profile crucial for cell identity and physiology.

### Relationship between gene expression and histone modifications in melon

**Fig. 2:**
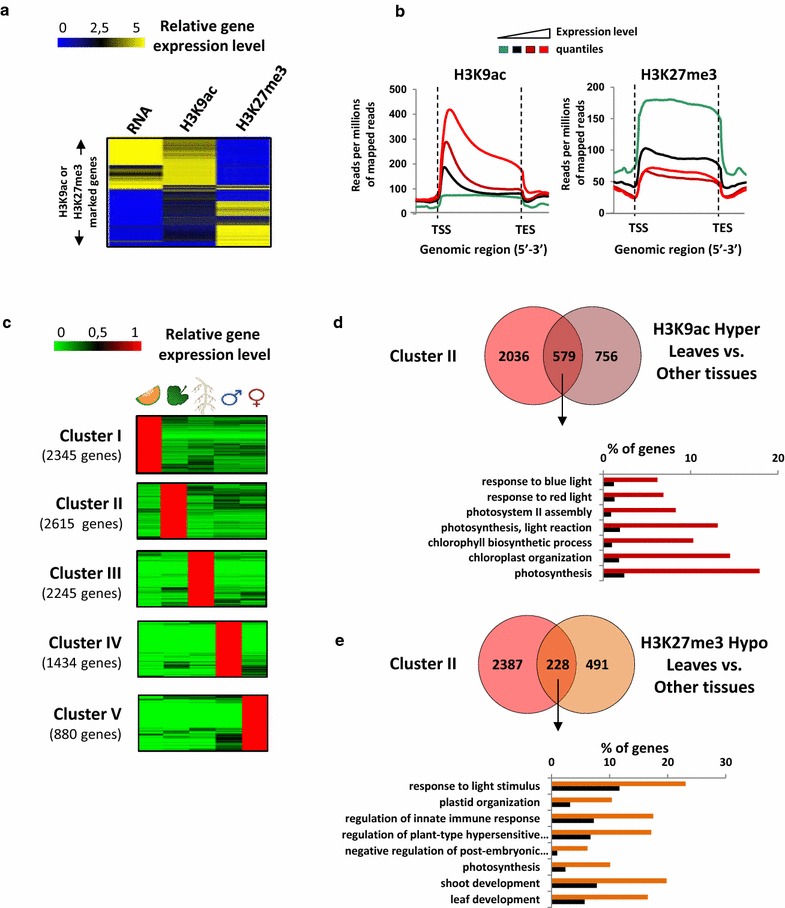
Correlation of H3K27me3 and H3K9ac with gene expression level and organ-specificity in leaves. **a** Correlation between H3K27me3, H3K9ac and gene expression. Genes specifically marked with H3K9ac or H3K27me3 in leaves were clustered based on the log2 value of their normalized tag densities of H3K9ac, H3K27me3 and RNA-seq data. Genes displaying the highest enrichment for H3K27me3 and H3K9ac were used with their corresponding gene expression levels in a heat map. **b** Correlation of between H3K27me3, H3K9ac and gene expression level. All the melon protein-coding genes were divided in 4 quantiles according to their gene expression levels (lowest and highest expression level corresponding to *green* and *red*, respectively). For each quantile the number of H3K27me3 and H3K9ac mapped reads was averaged and plotted along the gene body and 2-kb region flanking the TSS or the TES. **c** Gene clustering analyses based on organ-specific expression. Genes were sorted depending on their expression in each organ. 5 clusters, each one corresponding to an organ, are illustrated and numbered from 1 to 5. Number of genes of each cluster is also indicated. **d** Correlation of hyper-H3K9ac with tissue-specific gene activation. Venn diagram illustrating the genes whose high and tissue-specific expression is correlated with their tissue-specific hyper-acetylation (Overlapping set of genes). A Chi-squared test was done and confirmed the observed correlation. For this set of genes, GO terms of biological processes are shown below. **e** Correlation of hypo-H3K27me3 with tissue-specific gene activation. Venn diagram illustrating the genes whose high and tissue-specific expression is correlated with their tissue-specific hypomethylation (Overlapping set of genes). A Chi-squared test was done and confirmed the observed correlation. For this set of genes, GO terms of biological processes are shown below. *p* values for each enriched class are presented in Additional file 9: Table VIII

To connect H3K9ac and H3K27me3 histone marks with gene expression, we generated and integrated RNA-seq data from the five different organs. Firstly, we confirm that H3K9ac and H3K27me3 in *Cucumis melo* are, respectively, associated with gene expression and gene repression (Fig. 2a). Secondly, in order to determine whether the level of acetylation or methylation could be correlated with gene expression, we divided all the protein coding genes into four quantiles based on their expression levels and plotted their H3K9ac or H3K27me3 profile (Fig. 2b). We observed that H3K9ac occupancy increases with expression level, while H3K27me3 showed the opposite pattern, where it displayed a high enrichment in the lowest-expressed genes but it remained unresponsive to intermediary and high expression levels. This results suggest that in *Cucumis melo* the more a gene is marked by H3K9ac and H3K27me3, the more it will be expressed and repressed, respectively (Fig. 2b).

To confirm the relationship between gene expression and organ-specific landscape of H3K9ac and H3K27me3, we identified genes specifically expressed in each organ (Fig. 2c; Additional file 5: Table IV) and we compared them with organ-specific H3K9ac marked genes. Interestingly, in the case of leaves, we found that a significant part of genes specifically hyperacetylated (43%) and hypomethylated (32%) are specifically expressed in this organ (Fig. 2d, e). Gene ontology analyses of these genes showed a significant enrichment in photosynthesis and response to light. In the case of roots, flowers and fruits we observed the same tendency, suggesting that both H3K9ac and H3K27me3 controlled organ-specific gene expression (Additional file 1: Figure S13).

### Female and male flower-specific epigenome landscapes

**Fig. 3:**
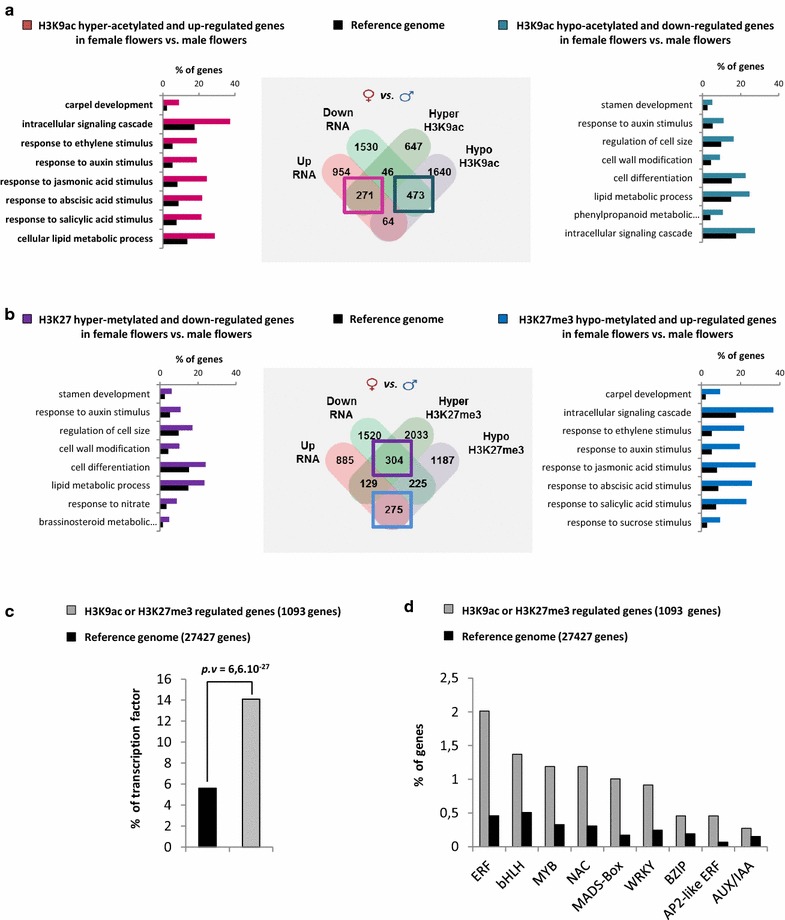
H3K9ac and H3K27me3 differentially marked genes between female and male flowers. **a** Venn diagram showing the relationship between H3K9 hyper-acetylated or H3K9 hypo-acetylated genes and up- or down-regulated genes in females flowers compared to male flowers (*middle panel*). Hyper-acetylated genes are predominantly up-regulated and hypo-acetylated genes are down-regulated, as highlighted in the *pink* and *blue boxes* respectively. Gene ontology analysis (GO) of the genes in the *pink* and *blue boxes* are shown in the *left panel* and the *right panel* respectively. Histograms of the values highlight the enrichment of genes compared to the reference. **b** Venn diagram showing the relationship of H3K27 hyper-methylated or H3K27 hypo-methylated genes and up- or down-regulated genes in females flowers compared to male flowers (*middle panel*). Hyper-methylated genes are predominantly down-regulated and hypo-acetylated genes are up-regulated, as highlighted in the *purple* and *blue boxes* respectively. Gene ontology analysis (GO) of the genes in the *purple* and *blue boxes* are shown in the *left panel* and the *right panel* respectively. Histograms of the values highlight the enrichment of genes compared to the reference genome (*black bars*). *p* values for each enriched class are presented in Additional file 9: Table VIII. **c** Percentage of transcription factors among the H3K9ac and H3K27me3 differentially regulated genes between male and female flowers. **d** Percentage of different known transcription factor families among the H3K9ac and H3K27me3 differentially regulated genes between male and female flowers

Beyond elucidating general aspects of the melon epigenome, the analyses we performed so far evidenced how H3K27me3 and H3K9ac organ-specific distributions can be crucial to study gene expression regulation and organ identity. Thus, we directed epigenomic and transcriptomic analyses toward the elucidation of female and male flower-specific epigenome landscapes and their role in sex determination. To this end, we integrated RNA-seq and, H3K9ac and H3K27me3 ChIP-seq data of male and female flowers in order to identify genes that are epigenetically regulated in a sex-specific manner (Fig. 3; Additional file 6: Table V). We observed that 22% of genes that are hypoacetylated in female flowers compared to male flower are downregulated and only 3% of the set of hypoacetylated genes are upregulated (Fig. 3a). Regarding hyperacetylated genes, we also found that a significant proportion of them (28%) are upregulated in female flowers compared to male flower and only 5% are dow-regulated. Furthermore, gene ontology analysis of female-specific epigenetically regulated genes showed enrichment in biological processes such as carpel development, response to ethylene and response to jasmonic acid. For male-specific epigenetically regulated genes, we observed enrichment in biological processes such as, stamen development and lipid metabolic process (Fig. 3a). The same analyses were performed for hypo- and hyper-methylated genes, obtaining similar results and confirming that a significant portion of sex-specific genes is epigenetically controlled (Fig. 3b).

After analyzing the biological processes involving the sex-specific epigenetically regulated genes, we classified these genes based on their molecular function. We found enrichment for transporter activity, catalytic activity, oxidoreductase activity and interestingly, for transcription factor (TF) activity (Fig. 3c; Additional file 1: Figures S14–S18; Additional file 7: Table VI). Since transcription factors are the best described and commonly associated components of gene expression regulation, we further focused on them and grouped them according to their TF families (Fig. 3d). We found that the ethylene-responsive factor (ERF) family displayed the highest number of gene members, consistent with previous studies that reported the role of ethylene and ethylene transduction pathways in sex determination in the cucurbitaceae family (22). In addition, we found other TF families such as Zinc Finger (17 genes), basic Helix-Loop-Helix (15 genes), MYB (13 genes) and NAC families (13) (Fig. 3d). Interestingly, we also detected 11 genes belonging to the MADS-box TF family. Several members of this family are conserved master regulators of flower ontogenesis [76–78]. When we assessed the identity of these 11 genes, we observed that all of them are potential homologs of different *Arabidopsis* genes that are part of the ABC model of flower development (Additional file 7: Table VI).

Altogether, these results highlight the organ-specificity of H3K27me3 and H3K9ac as well as its role in the regulation of key genes encoding transcription factors, which regulate processes such as ethylene response and flower development.

### Carpel and stamen epigenetic landscapes highlight sex-specific chromatin states of transcription factor genes

**Fig. 4:**
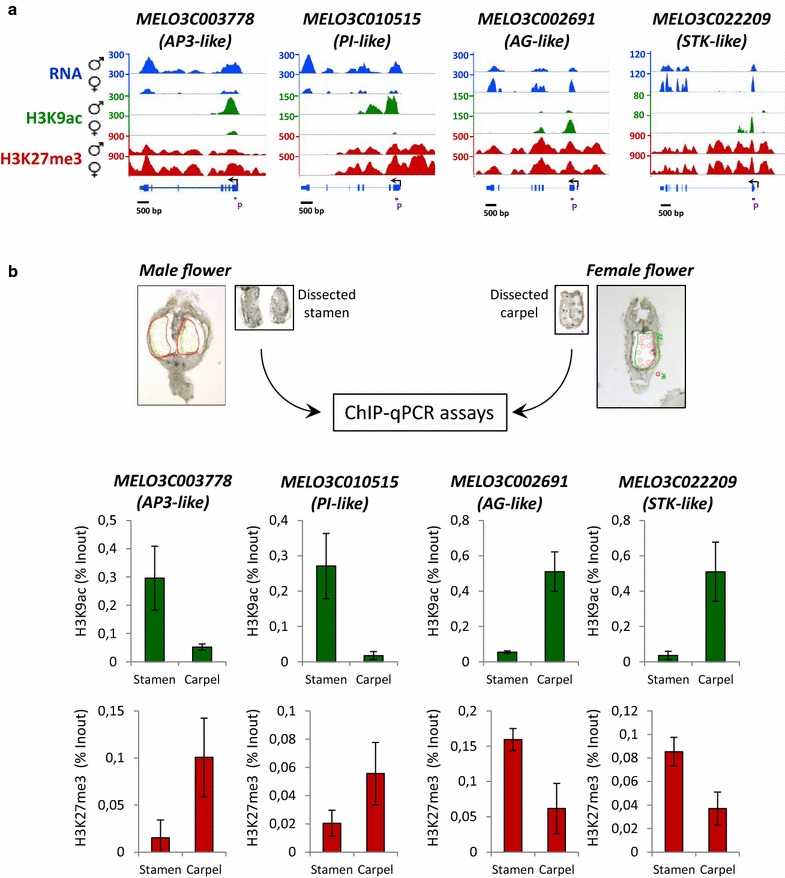
Carpel and Stamen epigenetic analysis of melon transcription factors. **a** H3K9ac and H3K27me3 profiles and RNA levels of four transcription factors in female and male flowers. Two TFs specific of the male flowers (*MELO3C003778* and *MELO3C0100515*) and two specific of the female flowers (*MELO3C002691* and *MELO3C022209*) were selected for epigenetic profiling in isolated carpels and stamens of melon flowers. The distributions of peaks and RNA reads obtained in our genome-wide analyses are plotted above the genome model for each TF. **b** Stamens and carpels of melon flowers were dissected by Laser Microdissection (LMD) and ChIP-qPCR assays were performed with anti-H3K9ac and anti-H3K27me3 antibodies. The *upper panels* show an example of dissections performed with the LCM technique. The *bar charts* represent the qPCR assays on immune-precipitated DNA using primers annealing a neighboring sequence to the TSS as indicated in *panel A* (*purple bar*)

Since sex determination in melon and other cucurbits relies on the selective regulation of carpel and stamen development, we analyzed in detail some melon TFs, which are potential homologs of *Arabidopsis* genes involved in the control of sexual organs development. For such genes, we first assessed the genic distribution of H3K27me3, H3K9ac and RNA-seq reads in male and female flowers (Fig. 4a). According to previous studies in *Arabidopsis*, stamen development relies on the expression of *APETELA3* (*AP3*)*, PISTILATA* (*PI*) and *AGAMOUS* (*AG*), whereas carpel development depends on the expression of ovule-specific genes such as *SEEDSTICK* (*STK*) and *SHATTERPROOF 1* (*SHP1*) [78]. As expected, when we analyzed both H3K9ac and H3K27me3 epigenetic landscapes of stamen identity genes together with their expression, we found that they are hyperacetylated, hypomethylated and highly expressed in the male flower when compared to the female flower. Carpel-promoting genes, however, presented an enrichment of H3K9ac and high gene expression in comparison to male flowers, but no significant differences were observed for H3K27me3, which may result from the cell heterogeneity of the sample or from the cooperative action of additional histone repressive marks.

Tissue heterogeneity is an important variable that limits the interpretation of ChIP-seq data and the identification of epigenetic events occurring in a specific cell population. This is particularly true in this context of flower development, where it was reported that organ identity relies on the whorl-specific expression of MADS-box genes [76–78]. We therefore decided to overcome such limitation, by performing an H3K27me3 and H3K9ac ChIP-qPCR in isolated carpels and stamens. We isolated carpels from female and stamens from male flowers through the Laser Capture Microdissection (LCM) technique [79] and performed ChIP using H3K27me3 and H3K9ac antibodies. The immunoprecipitated chromatin was then amplified using primers annealing a neighboring sequence to the TSS of *AP3, PI*, *AG*, *STK*-*like* genes; which corresponded to the highest peak of H3K9ac observed along the gene body. A negative control, *MELO3C115188*, that does not display any H3K9ac or H3K27me3 signals was also used (Additional file 1: Figure S19). In all cases, the obtained ChIP-qPCR results were consistent with the ChIP-seq data previously generated from entire flowers; however, the differences observed between stamen and carpel are more evident, presenting at least a fold change of 2.3. This allowed us to observe clear differences in H3K27me3 occupancy between male and female organs (Fig. 4b).

Altogether, these results indicate that H3K27me3 and H3K9ac target MADS-box TFs regulating carpel and stamen identity and are presented in a sex-specific manner, thereby suggesting the role of these two epigenetic marks in the regulation of their expression. Furthermore, these results showed how tissue homogeneity could represent an advantage for the elucidation of cell-specific epigenetic landscapes in melon and other plant species.

### Role of *CmACS11* and *CmWIP1* genes in sex-specific epigenome acquisition

**Fig. 5:**
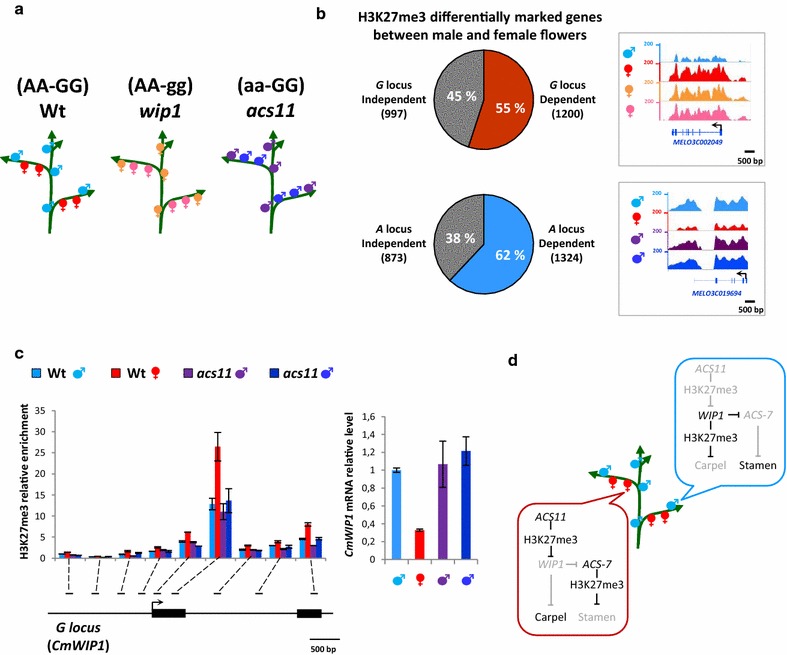
H3K27me3 genome-wide landscape in sex determination mutants. **a** Schematic representation of the different types of flowers used for H3K27me3 profiling in wild-type plants (AA-GG), *wip1* mutant (AA-gg) and *acs11* mutant (aa-GG) by ChIP-seq assays. In wild-type melon plants, flower sex is determined by their position on the inflorescence: flowers formed on nodes are male (*light blue*), then on each ramification, the first three flowers formed are female (*red*), and the next ones are male. Wild-type male and female flowers were compared to flowers occupying the same position on the inflorescence from the *g* (*orange* and* pink*) and *a* (*purple* and* blue dark*) mutant backgrounds. **b** Proportion of H3K27me3 changes between male and female wild-type flowers controlled by the *G* or *A* locus. To determine *G*-locus-dependent changes, a Venn diagram (Additional file 1: Figure S17) was first generated by comparing differential H3K27me3 deposition in wild-type male (*light blue*) and female flowers (*red*) with differential H3K27me3 deposition in wild-type male (*light blue*) and *g* mutant female flowers in the same position (*orange*). *A*-locus-dependent changes were determined in the same way by comparing differential H3K27me3 deposition in wild-type male (*light blue*) and female flowers (*red*) with differential H3K27me3 deposition in wild-type female flowers (*red*) and *a* mutant male flowers in the same position (*dark blue*). Proportions of *G* or *A* dependent or independent changes were then represented as a pie chart (*upper left panel* and *bottom left panel*, respectively). One example of H3K27me3 *G*-locus-dependent gene (*upper right panel*) and one example of H3K27me3 *A*-locus-dependent gene (*bottom down panel*) illustrate the H3K27me3 changes observed in the different ChIP-seq datasets. **c** *A*-dependent regulation of the *G* locus via H3K27me3 deposition. ChIP-qPCR with an anti-H3K27me3 antibody was performed in Wt and *a* mutant flowers using primers covering the entire G locus (*left panel*). Expression analysis of the *G* locus was performed on the same plant material by RT-qPCR assays (*right panel*). **d** Model illustrating the role of *A* and *G* locus as master regulators controlling sex-specific epigenome acquisition

We previously showed that sex determination in melon relies on the interplay between alleles of three sex determination genes, *M*, *G* and *A* [47, 49–51]. In monoecious melon, most of the flowers are male and female flowers develop at the youngest nodes of the growing vines (Fig. 5a). Male flowers result from the expression of the male promoting gene, *CmWIP1*. Female flowers develop on branches expressing *CmACS11*, which represses the expression of *CmWIP1*. Androecious plants result from a loss-of-function of *CmACS11* leading to expression of *CmWIP1* in all flowers on a plant. Gynoecious plants are obtained by inactivation of *CmWIP1* function [47, 51] (Fig. 5a).

To bring new insight on the role of these sex-determining genes in establishing epigenetic signatures important for sex determination in melon, we investigated the H3K27me3 genome-wide landscape in male and female flowers of wild-type monoecious melon and two androecious and gynoecious isogenic lines, carrying a loss-of-function mutation in *CmACS11* and *CmWIP*-*1,* respectively.

To determine the proportion of genes whose differential epigenetic state between male and female flowers can depend on *CmACS11* and *CmWIP*-*1*, we compared male and female flowers from the wild type with flowers occupying the same position on the inflorescence from androecious and gynoecious sex transition mutants, respectively (Fig. 5b; Additional file 1: Figures S20–S22). The gene lists generated by these comparisons are illustrated in a Venn diagram, where the overlapping genes correspond to *CmWIP1*—(Additional file 1: Figure S20B) and *CmACS11*-related (Additional file 1: Figure S20C) H3K27me3 changes linked to sex identity. We observed that 54% (1200/2197) of sex-specific regulated gene was under the control of the *G* locus and that 60% (1324/2197) was under the control of the *A* locus (Fig. 5b). Altogether, these data suggested that *CmACS11* and *CmWIP*-*1* are two master regulator controlling sex-specific epigenome acquisitions.

The androecious gene *CmACS11* plays central role in sex determination as it controls the expression the gynoecious gene *CmWIP1* [51]. Knowing from this study that *CmWIP1* is epigenetically regulated through histone methylation (Additional file 1: Figure S23), we predicted that *CmACS11* represses the expression of the *CmWIP1* by modifying its methylation status. To test this hypothesis, we analyzed the H3K27me3 status of *CmWIP1* in flowers that do or do not express *CmACS11* and in flowers impaired in *CmACS11* function. We performed an H3K27me3 ChIP followed by qPCR using 9 different primers covering the entire *CmWIP1 gene*. As predicted, we observed that *CmWIP1* is hypermethylated in female buds that express *CmACS11* but not in male buds that do not express *CmACS11*. The hypermethylation of *CmWIP1* is also lost in *CmACS11* loss of function mutant (Fig. 5c). The hypomethylation correlates with the expression *CmWIP1* and the hypermethylation with the non-expression of *CmWIP1*. Based on this, we concluded that the *A* gene represses the expression of *CmWIP1* via deposition of H3K27me3.

## Discussion

Deposition of chromatin modifications is instrumental for the concerted expression or repression of thousands of genes, thereby allowing the establishment of organ or tissue specific transcriptomes [10–13]. H3K9ac and H3K27me3 are among the main chromatin marks involved in the control of gene expression [5, 20, 30–33]. In this study, we generated genome-wide and organ-specific maps of H3K27me3 and H3K9ac in melon, thereby generating a repertoire for the elucidation of mechanisms controlling the expression of the melon genome. This work will provide new information for the analysis of melon development or several agricultural traits, that have until now been mainly studied via classical genetic approaches. The distributions of H3K9ac and H3K27me3 in the melon genome displayed common chromosomal and genic distributions when compared to other plant species such as *Arabidopsis* [14–17], maize [19, 20] and rice [21–23]: H3K27me3 and H3K9ac were mainly enriched at the chromosome arms, resembling the distribution of genes, but were confined to the gene body and not spread along intergenic regions. Within the gene body, H3K27me3 distributes uniformly from the TSS to the TES, whereas H3K9ac is mainly enriched at the TSS.

As described in other plant species, we observed that H3K9ac and H3K27me3 marks display opposite correlation with gene expression. Interestingly, we found a direct proportionality between H3K9ac and gene expression levels, thereby suggesting the crucial role of H3K9ac in gene expression regulation and fine-tuning. In contrast, H3K27me3 did not display an evident proportionality with gene expression, being unresponsive to intermediate gene expression levels as it has been observed for barley [25] and maize [19]; however, it was clearly enriched at the lowest-expressed genes, suggesting that this histone mark is involved in the full repression of genes rather in the fine-tuning of their expression.

Beyond their role in gene expression regulation, we observed that both H3K9ac and H3K27me3 are important in the control of organ identity and development, thus mediating the formation of different cell types and functions from a single genome. A considerable portion of H3K9ac and H3K27me3 target genes was expressed in an organ-specific manner, the differential and unique deposition of these two histone marks in each organ, creates an epigenetic landscape on genes that is crucial for its metabolic needs and physiology.

In the case of flowers, female-specific H3K9 hyper-acetylated and H3K27 hypo-trimethylated genes are involved in carpel and flower development as well as in several other bioprocesses previously reported to play a role in flower feminization in cucurbits. Such is the case for the hormone ethylene, whose synthesis and perception have important roles in development of female unisexual flowers. The expression of *CmACS7* and *CmACS11,* two enzymes that are part of the ethylene biosynthesis pathway, and *CsETR1*, one of the reported ethylene receptors, have been already placed in the regulatory network promoting female flower development in cucurbits [47, 49–51, 80–83]. On the other hand, other genes related to jasmonic acid response were specifically modified with active marks in the female flower. Consistent with this, jasmonic acid (JA) was also shown to be associated with sex determination in maize [84]. Specifically, downregulated genes (H3K9 hypo-acetylated and H3K27 hyper-trimethylated) in the female flower pointed out bioprocesses such as lipid metabolism, which has been previously reported to play a role in pollen-tube growth and pollen-stigma interactions [85].

When we assessed the molecular function of sex-specific epigenetically regulated genes, we found enrichment for transcription factor, DNA and nucleotide binding activities. We, therefore, categorized these TFs according to their corresponding protein families and found that the majority of them belong to the ethylene-responsive factors [86–88]. These results suggest that histone modifications regulate ethylene transduction pathway in melon as it has previously reported in Arabidopsis [89].

Interestingly, we also found genes belonging to the MADS-box TF family, which are well recognized for their important role in flower whorl identity and is in concordance with previous reports regarding the regulation of *AP1, AG* and *SEP3* by the histone demethylase REF6 that removes H3K27me3 [90, 91], and polycomb-dependent regulation of *AG* [92, 93].

The elucidation of epigenetic control of the class C genes for the development of unisexual flowers demands the assessment of the whorl-specific epigenetic state of carpel- and stamen-related identity of MADS-box genes. Tissue heterogeneity has been considered a persistent constraint for the identification of epigenetic events occurring in a specific cell population thus, masking the natural complexity of gene expression regulation. So far in the plant kingdom, few technical improvements to this limitation have been reported, especially due to the complexity of performing ChIP on small samples. In this study, we provide the first protocol to overcome such limitation that can be transferred to any plant species and demonstrate that epigenetic changes that are undetectable with standard methods using whole organs can be identified by using purified cell populations. By this method, we also demonstrated that stamen—(i.e., *PI* and *AP3*) and carpel-related (i.e., *STK*) identity genes present a contrasting H3K27me3 and H3K9ac landscapes. This suggests that the sex determination genes recruit histone modifiers to target organ identity genes in the sexual whorls of the flower, leading to unisexual flower development.

In the cucurbit sex determination pathway, the gynoecious gene, *CmWIP1*, plays a central role in unisexual flower development. From this analysis, we showed that CmACS11 represses *CmWIP1* expression by inducing H3K27me3 at this locus (Fig. 5d). This data suggest that the local production of ethylene via CmACS11 in companion cells is able to activate histone methyltransferase to inhibit *CmWIP1* expression in a defined spatio-temporal manner.

Detailed knowledge about spatio-temporal gene expression and epigenetic regulation dynamics is pivotal for a comprehensive understanding of development of unisexual flowers. In species recalcitrant to plant transformation, such as melon, this has been hampered mainly by difficulties in isolating sufficient amounts of tissues from distinct organ primordia for chromatin analysis. In this context, using laser capture micro-dissection, we succeeded in generating high-quality chromatin, from dissected carpel and stamina primordia, from unisexual flowers, and discovered that the sex determination genes recruit histone modifiers to orchestrate unisexual flower development in monoecious species. Genome-wide analyses of chromatin modifications in the dissected reproductive organs from unisexual and hermaphrodite flowers and from key histone modifier mutants will likely to bring new insight on the epigenetic control of unisexual flower development.

## Methods

### Plant material and growth conditions

Monoecious melon (*Cucumis melo*) cultivar charentais (*Cucumis melo* L. subsp. *melo* var *cantalupensis*) were used for H3K9ac and H3K27me3 genome-wide descriptive analyses. For each replicate, 7-day-old seedlings were transferred to individual pots and incubated in a growth chamber for 30 days (long day conditions, temperature: 27 °C (day) and 21 °C (night), relative humidity: 60%). Young male and female flowers (3 mm length; Developmental stage 8 [52]) were collected, organized according to their location in the plant (i.e., main stem or branches) and conserved at −80 °C for epigenomic and transcriptomic analyses.

For the determination of *a*- and *g*-related epigenetic changes, we used two families of EMS-treated *C. melo* lines carrying missense mutations in *CmWIP1* and in *CmACS11*, both previously reported to lead to gynoecy [47] and androecy [51], respectively. Germination, growth and flower collecting conditions were the same to the previously described for the monoecious WT plants.

### Gene expression analysis

Total RNA were extracted from leaves, root, fruit, male and female flowers using the Nucleospin RNA kit (Macherey–Nagel), according to the manufacturer’s instructions. For RT-QPCR analyses, first-strand cDNA was synthesized from 1 μg of total RNA using Improm-II reverse transcriptase (A3802, Promega) according to the manufacturer’s instructions. 1/25th of the synthesized cDNA was mixed with 100 nM of each primer and LightCycler^®^ 480 Sybr Green I master mix (Roche Applied Science) for quantitative PCR analysis. Products were amplified and fluorescent signals acquired with a LightCycler^®^ 480 detection system. The specificity of amplification products was determined by melting curves. ACT2 was used as internal control for signals normalization. Exor4 relative quantification software (Roche Applied Science) automatically calculated relative expression level of the selected genes with algorithms based on ΔΔCt method. Data were from duplicates of at least two biological replicates. The sequences of primers can be found in Additional file 8: Table VII.

For RNA-seq analysis, libraries were synthetized using NEBNext Ultra Directional RNA library Preparation Kit (NEB) according to the manufacturer’s instructions. Two biological replicates were run for each tissue. Raw reads from RNA-Seq were first adaptor trimmed and quality filtered using Trimmomatic [94]. Filtered reads were mapped to the *Cucumis melo* genome v3.5.1 using TopHat v2.0.9 [95]. Transcript quantification was derived using Cufflinks v2.2.0 [96]. CuffDiff is used for differential expression analysis (*p* value 0.05; statistical correction: Benjamini Hochberg; FDR: 0.05). A cutoff of 0.5 fold up- or down-regulation has been chosen to define significant differential expression. CummeRbund v2.0.0 was used for visualization of differential analysis [97]. Functional annotation using GO terms was performed using the Plant MetGenMAP tool [75]. All *p* values are shown in Additional file 9: Table VIII.

### Chromatin immunoprecipitation experiments

ChIP assays were performed using anti-H3K9ac (Millipore, ref. 07-352) or anti-H3K27me3 (Millipore, ref. 07-449) antibodies, using a procedure adapted from Veluchamy et al. [17]. Briefly, after plant material fixation in 1% (v/v) formaldehyde, tissues were homogenized, nuclei isolated and lysed. Cross-linked chromatin was sonicated using a water bath Bioruptor UCD-200 (Diagenode, Liège, Belgium) (30 s on/30 s off pulses, at high intensity for 60 min). Protein/DNA complexes were immunoprecipitated with antibodies, overnight at 4 °C with gentle shaking, and incubated for 1 h at 4 °C with 50 μL of Dynabeads Protein A (Invitrogen, Ref. 100-02D). The beads were washed for 2 × 5 min in ChIP Wash Buffer 1 (0.1% SDS, 1% Triton X-100, 20mM Tris-HCl pH 8, 2 mM EDTA pH 8, 150 mM NaCl), 2 × 5 min in ChIP Wash Buffer 2 (0.1% SDS, 1% Triton X-100, 20 mM Tris-HCl pH 8, 2 mM EDTA pH 8, 500 mM NaCl), 2 × 5 min in ChIP Wash Buffer 3 (0.25 M LiCl, 1% NP-40, 1% sodium deoxycholate, 10 mM Tris-HCl pH 8,1 mM EDTA pH 8) and twice in TE (10 mM Tris-HCl pH 8, 1 mM EDTA pH 8). ChIPed DNA was eluted by two 15-min incubations at 65 °C with 250 μL Elution Buffer (1% SDS, 0.1 M NaHCO_3_). Chromatin was reverse-crosslinked by adding 20 μL of NaCl 5 M and incubated over-night at 65 °C. Reverse-cross-linked DNA was submitted to RNase and proteinase K digestion and extracted with phenol–chloroform. DNA was ethanol precipitated in the presence of 20 μg of glycogen and resuspended in 50 μL of nuclease-free water (Ambion) in a DNA low-bind tube.

For ChIP-qPCR experiments, fold enrichment of targets in ChIPed DNA relative to input was calculated from an average of three replicate qPCRs. The sequences of primers can be found in Additional file 8: Table VII. Positions of the amplified regions on the different loci are indicated in Fig. 5.

For ChIP-seq assays, 10 ng of IP or input DNA was used for ChIP-Seq library construction using NEB-Next Ultra II DNA Library Prep Kit for Illumina (New England Biolabs) according to manufacturer’s recommendations. For all libraries, twelve cycles of PCR were used. The quality of the libraries was assessed with Agilent 2100 Bioanalyzer (Agilent), and the libraries were subjected to high-throughput sequencing by Illumina Sequencing technology.

### Computational analysis of ChIP-seq

Preprocessing of sequencing reads for quality was performed using FASTQC (http://www.bioinformatics.babraham.ac.uk/projects/fastqc/). Filtering and trimming of reads was done using Trimmomatic with the following parameters: Minimum length of 36 bp; mean Phred quality score greater than 30; leading and trailing bases removal with base quality below 3; sliding window of 4:15. Using Bowtie, the remaining high-quality reads were mapped onto the *Cucumis melo* genome v3.5.1 with maximum mismatch of 1 bp. Unique mapping of reads was adopted. To determine the target regions of H3K9Ac ChIP-Seq, the model-based analysis of ChIP-Seq (MACS2) was adopted (Number of duplicate reads at a location: 1; Bandwidth: 300; mfold of 5:30; q-value cutoff: 0.05) [72]. For peak detection in H3K27me3 modified regions, we used SICER with the following criteria: Window size: 200, Gap size: 600 [98]. Alignment and tag-density were inspected with IGB. HOMER was used to associate peaks to nearby genes [99]. To cluster the H3K9Ac and H3K27me3 peaks, linear normalization and clustering of tag density with Density Array method (window size 50 bp; 2 kb flanking region of genes) was performed using SeqMINER [100]. Annotation of corresponding genes was done using melonomics resource (https://melonomics.net). Average profile of coverage along the genic region (between transcriptional start sites (TSS) and TES) along with the 2 kb flanking region was plotted using NGSplot in binning mode [101]. To identify differentially enriched histone modified sites in leaves, root, fruit and flowers, we used DiffReps with the following settings: Window size 1 kb; step size: 100 bp; *p* value: 0.0001; Statistical testing method: Chi-square method) [74].

### Chromatin immunoprecipitation of Laser capture microdissected primordia

Male and female flowers of 3 mm were collected on monoecious melon plants, fixed in 1% (v/v) formaldehyde for 15 min under vacuum, and the crosslink reaction was stopped by addition of glycine (130 mM final) for 5 min under vacuum. Fixed material was then rinsed with water, dried and snap-frozen in liquid nitrogen. In a cryostat, flowers were put and oriented at the bottom of an empty cryomold and covered by cryoprotector (Tissue-Tek^®^ O.C.T. Compound, Sakura^®^ Finetek, VWR, France) until frozen. After 10 min at −20 °C in the cryostat (Shandon Cryotome^®^ FSE, Excilone, France), samples were sectioned at −20 °C. To locate the targeted structures, sections were collected on glass slide and observed through the optical microscope of an XT^®^ Arcturus Technologies microdissection system (Excilone, France). The following 4 sections (30 µm thickness) containing 10 flowers were collected on Arcturus FRAME membrane (Excilone, France). Then, the frame was put to room temperature for the sections to melt on the membrane and a glass slide was added on top of the membrane. The whole system is then placed upside down in the microdissection system. The LCM process was carried out using the XT^®^ Arcturus Technologies microdissection system and software. Capture was performed using Arcturus CapSure^®^ LCM Macro Caps (Excilone, France). Carpels and stamens were identified using the 20X. FRAME Membrane Slides allowed the use of standard IR and UV lasers. UV laser was used to cut around the selected regions as IR laser was used to capture the selected regions on the macro caps without contaminating the sample with non-target-tissue. Efficiency of microdissection was evaluated by examining the CapSure^®^ Macro caps after capture and the tissue section remaining on the slide before and after lifting off the CapSure^®^ macro caps. To obtain enough material for downstream analysis, around 5 mm^2^ of captured area was collected on 3 or 4 caps per sample and stored at −80 °C. Tissues were then disrupted by grinding with mortar and pestle in liquid nitrogen. Cellular debris was then resuspended in 2 ml of nuclei isolation buffer (20 mM Hepes pH8; 250 mM sucrose; 1 mM MgCl2; 5 mM KCl; 40% Glycerol; 0,25% Triton X-100), filtered through a strainer with a 63 µm pore size. Nuclei integrity was assessed by Dapi coloration and visualization under an epifluorescence microscope. Next, the samples were centrifuged 10 min at 3500*g* and the pellet was resuspended in 130 µl of nuclei lysis buffer (50 mM Tris–HCl pH8; 10 mM EDTA). Chromatin was sonicated using Covaris (M220 Focused-ultrasonicator; DC 5%; PIP 75Watts; CPB 200; 20 min) in 130 µl microtubes (Covaris AFA tubes) and centrifuged 10 min at 16,000*g*. Supernatant was then diluted ten times with ChIP dilution buffer (1,1% Triton; 1,2 mM EDTA; 16,7 mM Tris–HCl pH8; 167 mM NaCl) before immunoprecipitation with H3K9ac or H3K27me3 antibodies overnight at 4 °C with gentle shaking, and incubated for 1 h at 4 °C with 50 μL of Dynabeads Protein A (Invitrogen, Ref. 100-02D). After several washes, immunoprecipitated DNA was then recovered using proteinase K digestion, reverse crosslink and phenol–chloroform extraction as previously described. Fold enrichment of targets in ChIPed DNA relative to input was calculated from an average of three replicate qPCRs. The sequences of primers can be found in Additional file 8: Table VII. Positions of the amplified regions on the different loci are indicated in Fig. 4.

## Additional files

**Additional file 1: Figure S1.** Pearson correlation between leaves and roots ChIP-seq replicates. The color scale represents the degree of correlation between replicates. **Figure S2.** Example of melon genomic regions showing ChIP-seq signals of biological replicates in leaves. Genes are represented in blue. Comparisons of peak positions and peak intensities illustrate the high correlation between the two biological replicates. **Figure S3.** Distribution of mapped reads for H3K27me3 (red shades) and H3K9ac (green shades) along the 12 melon chromosomes of leaves, roots, fruit, male and female flowers. Local peak densities of each epigenetic mark were plotted against the genetic distance (gray) and annotation of sense (dark blue) and antisense transcripts (light blue). **Figure S4.** Number of H3K9ac and H3K27me3 peaks identified in the different melon tissues. The computational methods MACS2 and SICER were used to determine H3K9ac and H3K27me3 target regions, respectively. **Figure S5.** H3K27me3 and H3K9ac distribution at the chromosome level in different melon organs. Distribution of H3K9ac (Green), H3K27me3 (*red*) and annotated genes (gray) are plotted along the chromosome 1. **Figure S6.** H3K9ac and H3K27me3 peaks distribution on chromosome 3 illustrating the enrichment of the marks in gene-rich regions located at the distal part of the chromosome. **Figure S7.** Boxplot showing differential peak length between H3K9ac and H3K27me3 in different melon tissues. **Figure S8.** Average tag density profile of H3K27me3 and H3K9ac along the gene body in different melon tissues. ChIP-Seq densities of equal bins were plotted along the gene body and 2-kb region flanking the TSS or the TES. **Figure S9-S12.** Tissue-specific H3K9 or H3K27me3 differentially marked genes. Venn diagrams of paired comparisons of each tissue vs. all the tissues are shown on the left. The central overlap corresponds to H3K9 or H3K27me3 specifically modified genes in each tissue. Gene ontology analysis of these subsets of genes is shown on the right. **Figure S13.** Correlation of hyper- H3K9ac or hypo-H3K27me3 with tissue-specific gene activation. Venn diagram illustrating the genes whose high and tissue-specific expression is correlated with their tissue-specific H3K9 hyper-acetylation or H3K27me3 hypo-methylation (Overlapping set of genes). A Chi-squared test was done for each comparison and confirmed the observed correlation. **Figure S14.** Molecular function analysis of H3K9ac or H3K27me3 differentially regulated genes in male and female flowers. The most significant enrichment is observed for the transcription factor activity category. **Figure S15-S18.** Molecular function and biological process classification of H3K9ac and H3K27me3 differentially regulated genes in male and female flowers. Bar charts represent the number of genes in each category for the molecular function (left) and the biological process (right). **Figure S19.** ChIP-qPCR assays on microdissected stamens and carpels of a genome region that do not display any H3K9ac or H3K27me3 signals in the ChIP-seq data. **Figure S20.** Analysis of H3K27me3 deposition in *a* and *g* mutant flowers. **A.** Schematic representation of the different type of flowers used for H3K27me3 profiling in wild-type plants (AA-GG), *wip1* mutant (AA-gg) and *acs11* mutant (aa-GG) by ChIP-seq assays. **B.** Comparisons of differential H3K27me3 deposition in wild-type male (light blue) and female flowers (*red*) with differential H3K27me3 deposition in wild-type male (light blue) and *g* mutant female flowers in the same position (orange). **C.** Comparisons of differential H3K27me3 deposition in wild-type male (light blue) and female flowers (*red*) with differential H3K27me3 deposition in wild-type female flowers (*red*) and *a* mutant male flowers in the same position (dark blue). **Figure S21.** Proportion of H3K27me3 changes between male and female wild-type flowers controlled by the *G* or *A* locus. Hypo- and hyper-methylated genes were analyzed separately. **Figure S22.** GO analysis of G- and A-H3K27me3 dependent genes. **Figure S23.** Browser view of the H3K27me3 ChIP-seq signals on the *G* locus in Wt flowers and *a* mutant flowers. Only exon 2 of the *G locus* is present in the pseudomolecule of the 12 chromosomes in the 3.5.1 melon genome version. Promoter and exon 1 of the G locus are present on another pseudomolecule that contains non-anchored BAC clones (identified as chromosome 0 in the 3.5.1 melon genome version).

**Additional file 2: Table I.** Number of aligned reads for each library.

**Additional file 3: Table II.** List of H3K9ac and H3K27me3 marked genes in leaves, root, fruit, male and female flowers.

**Additional file 4: Table III.** List of H3K9ac and H3K27me3 specifically enriched or depleted genes in leaves, root, fruit, male and female flowers.

**Additional file 5: Table IV.** List of specifically over-expressed genes in leaves, root, fruit, male and female flowers.

**Additional file 6: Table V.** List of H3K9ac and H3K27me3 regulated genes in flowers.

**Additional file 7: Table VI.** List of transcription factor epigenetically regulated genes in flowers.

**Additional file 8: Table VII.** List of primers used in this study.

**Additional file 9: Table VIII.** *p*-values list of GO analyses.
