# Supplementary material for: The quest for epigenetic regulation underlying unisexual flower development in Cucumis melo
Source: Epigenetics Chromatin. 2017 Jun 6;10:22. doi: 10.1186/s13072-017-0132-6 (PMC5460419; doi:10.1186/s13072-017-0132-6)

Additional file 1 : Figure S1

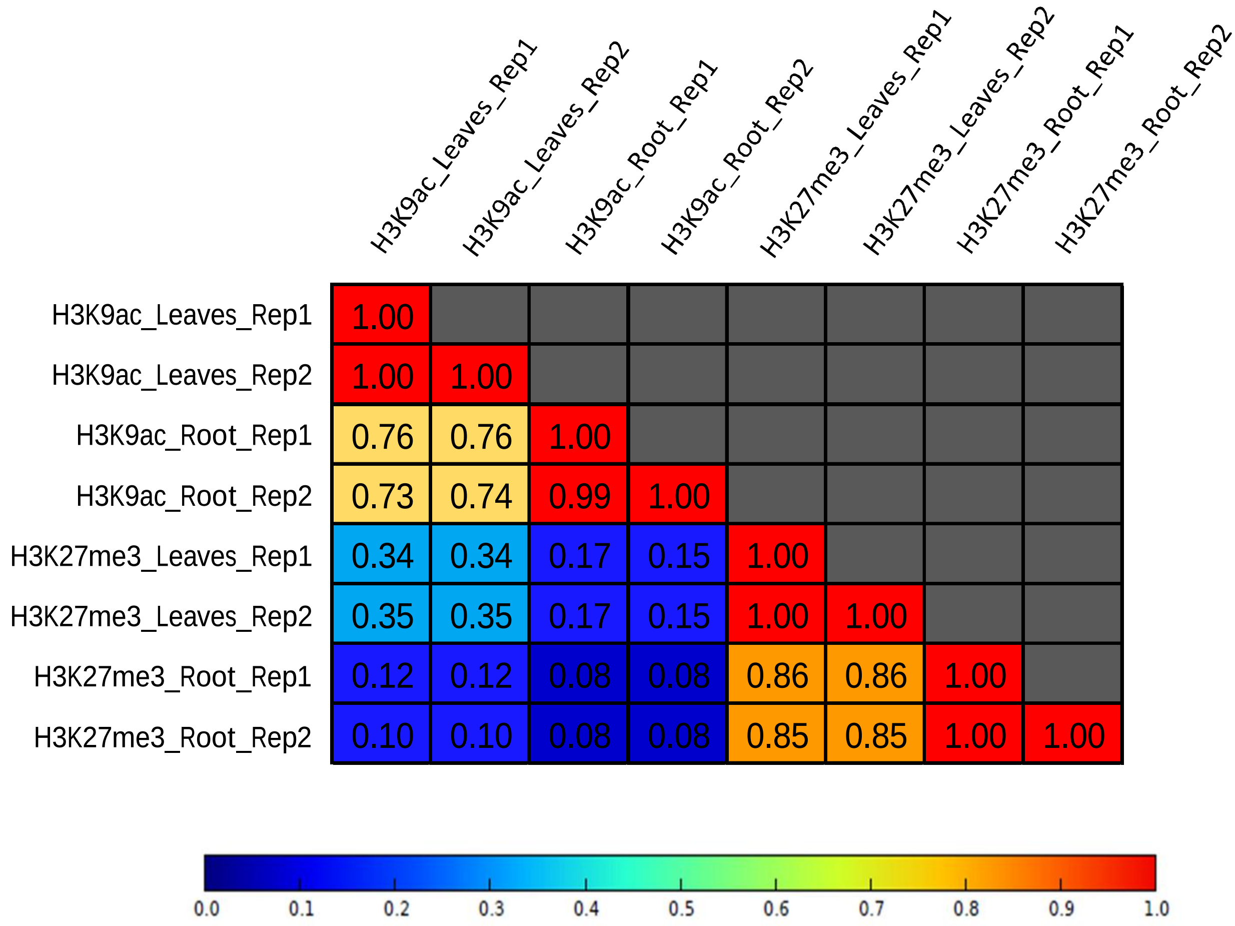

Additional file 1 : Figure S2

H3K9ac - Leaves

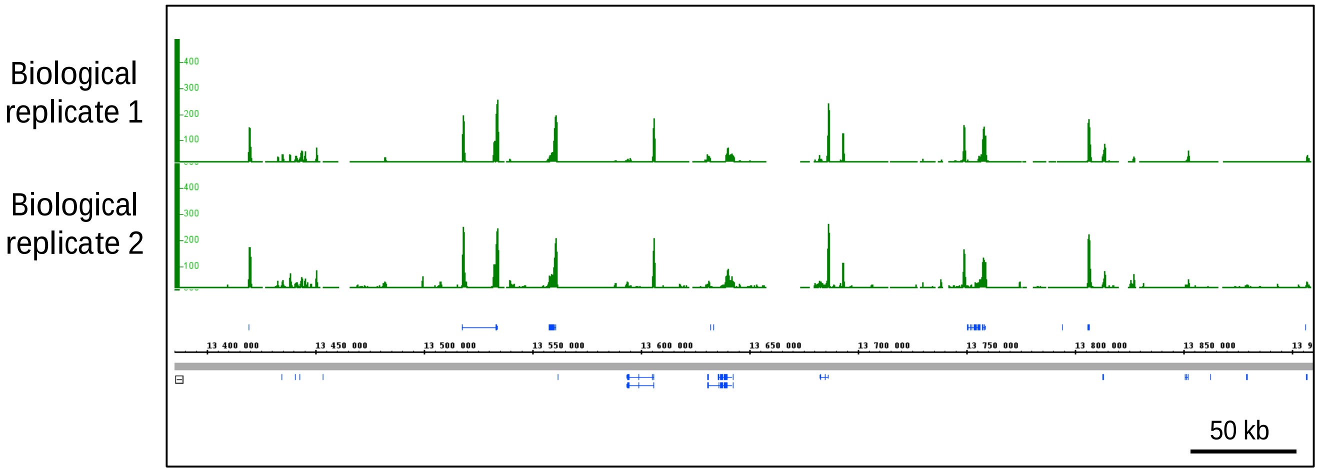

H3K27me3 - Leaves

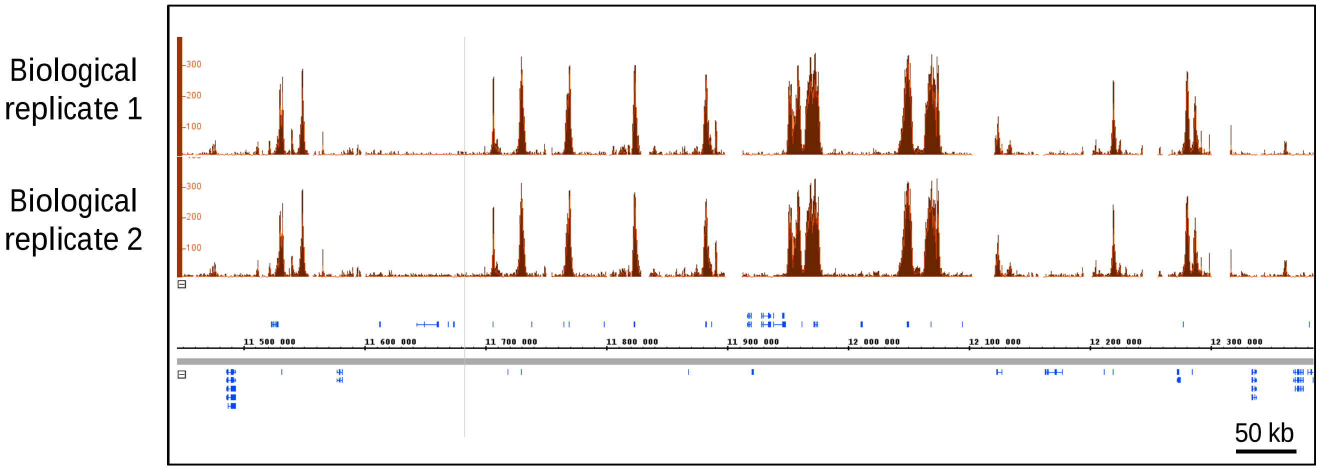

Additional file 1 : Figure S3

- H3K9ac Leaves
- H3K9ac Root
- H3K9ac Fruit
- H3K9ac Female flowers
- H3K9ac Male flowers
- Genes (+)
- H3K27me3 Leaves
- H3K27me3 Root
- H3K27me3 Fruit
- H3K27me3 Female flowers
- H3K27me3 Male flowers
- Genes (-)

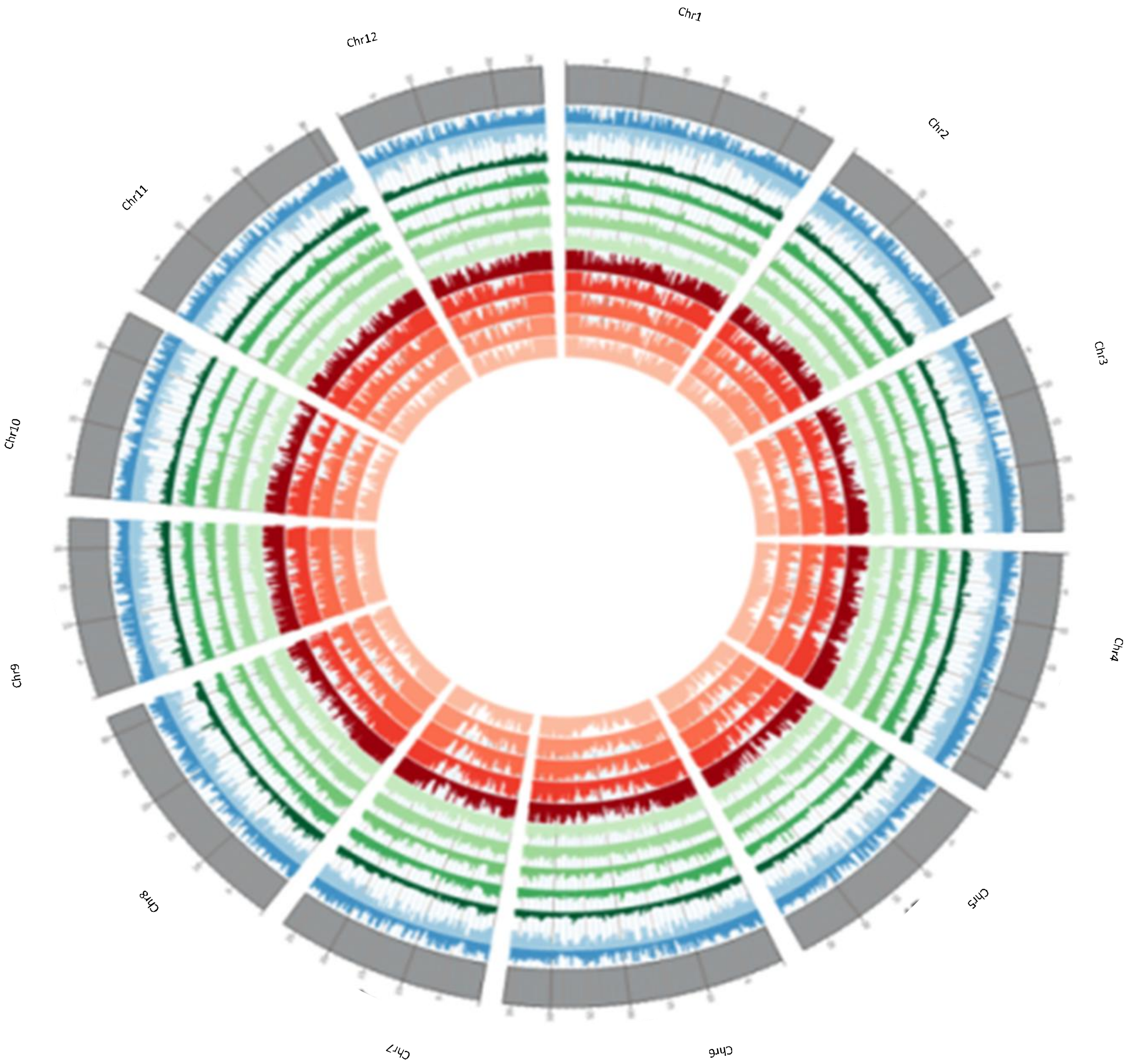

Additional file 1 : Figure S4

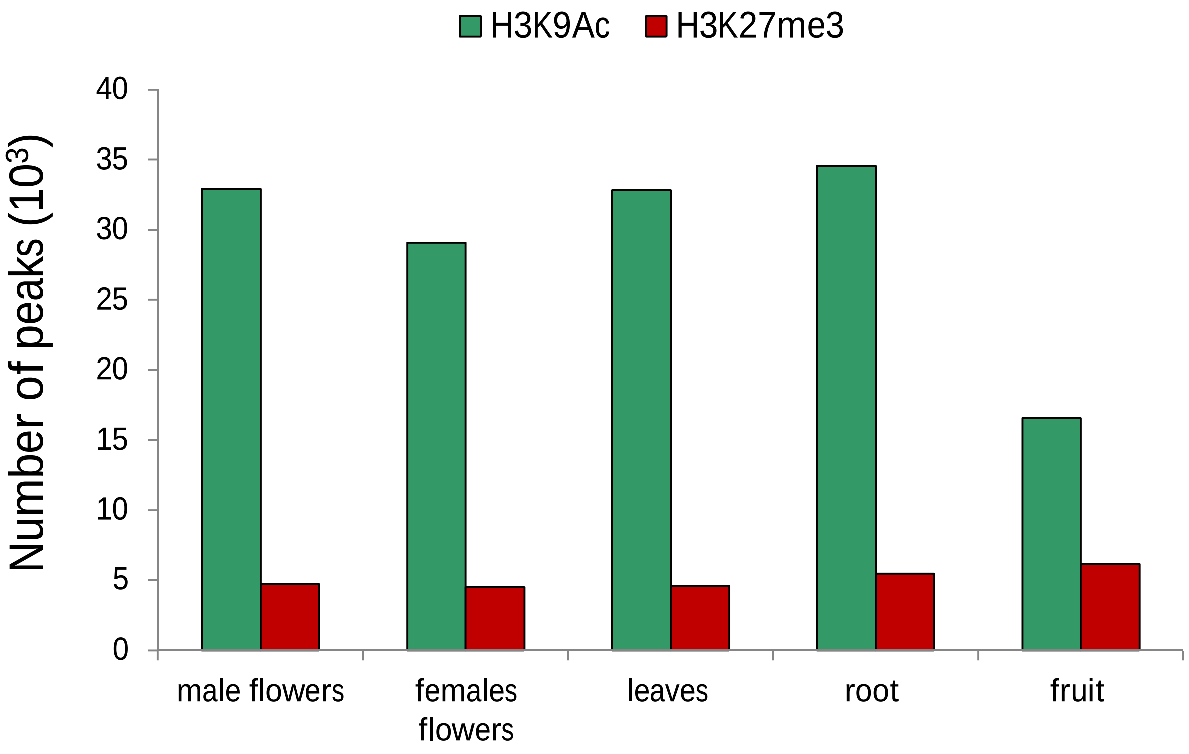

Additional file 1 : Figure S5

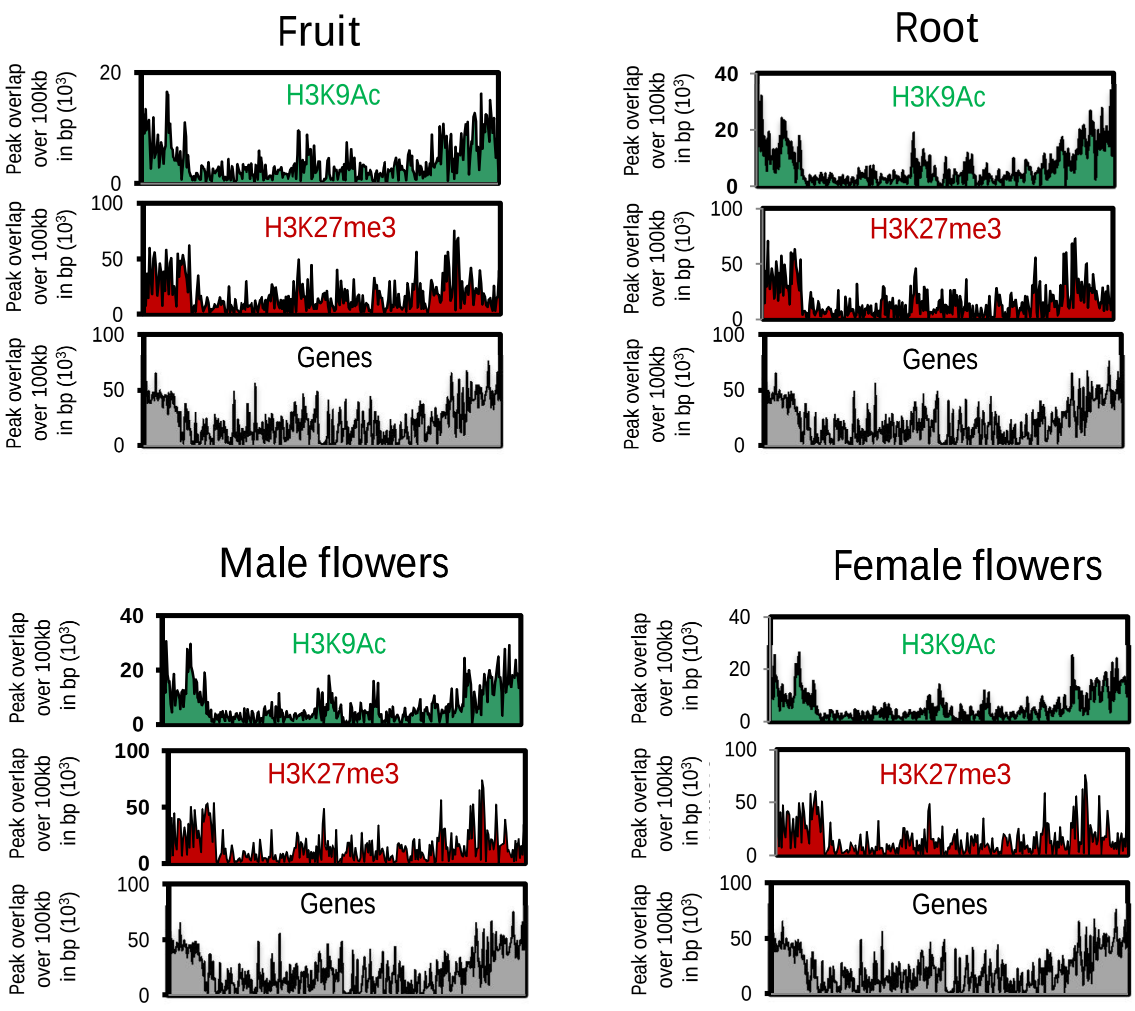

Additional file 1 : Figure S6

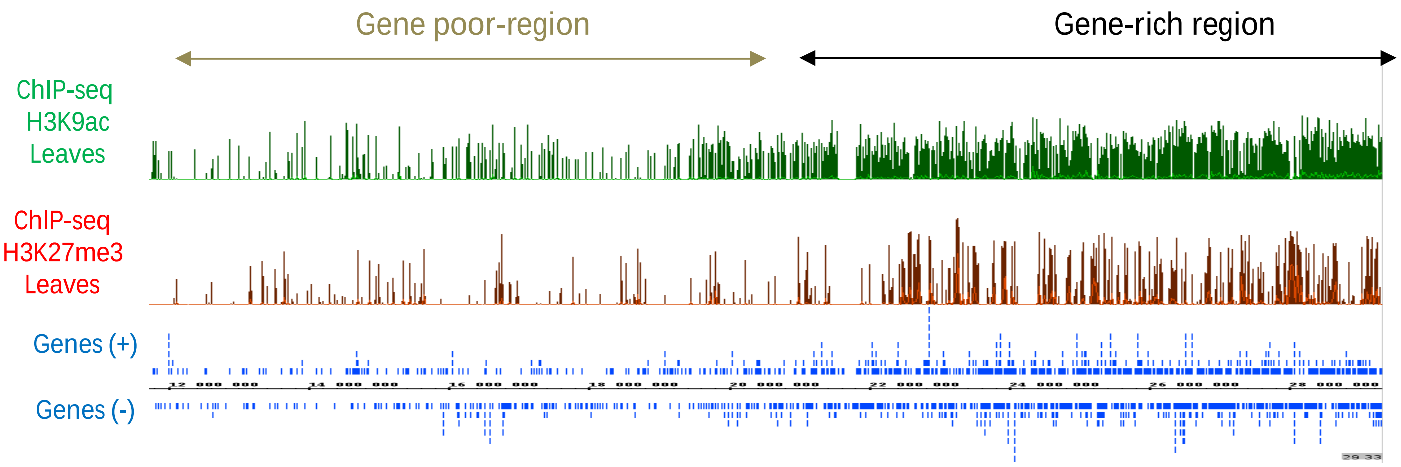

Additional file 1 : Figure S7

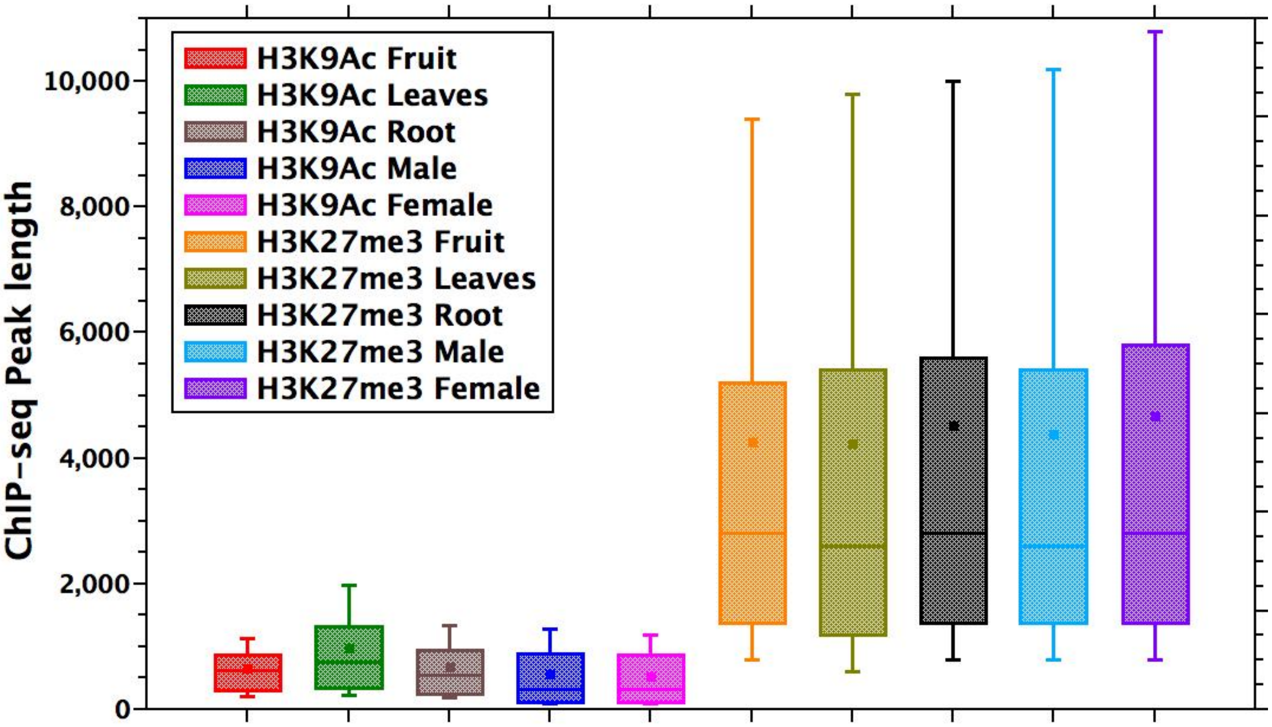

Additional file 1 : Figure S8

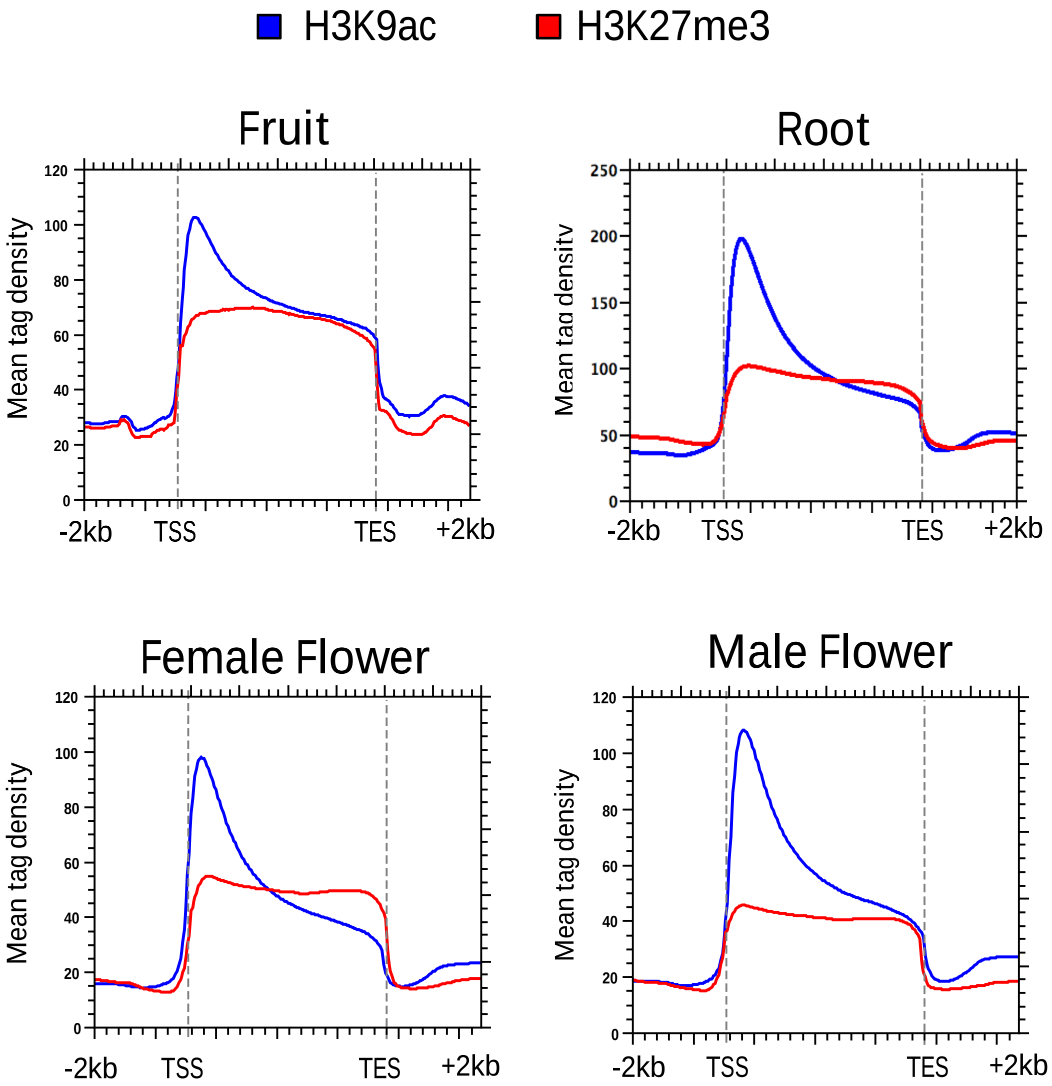

Additional file 1 : Figure S9

H3K9ac hyper-acetylated genes

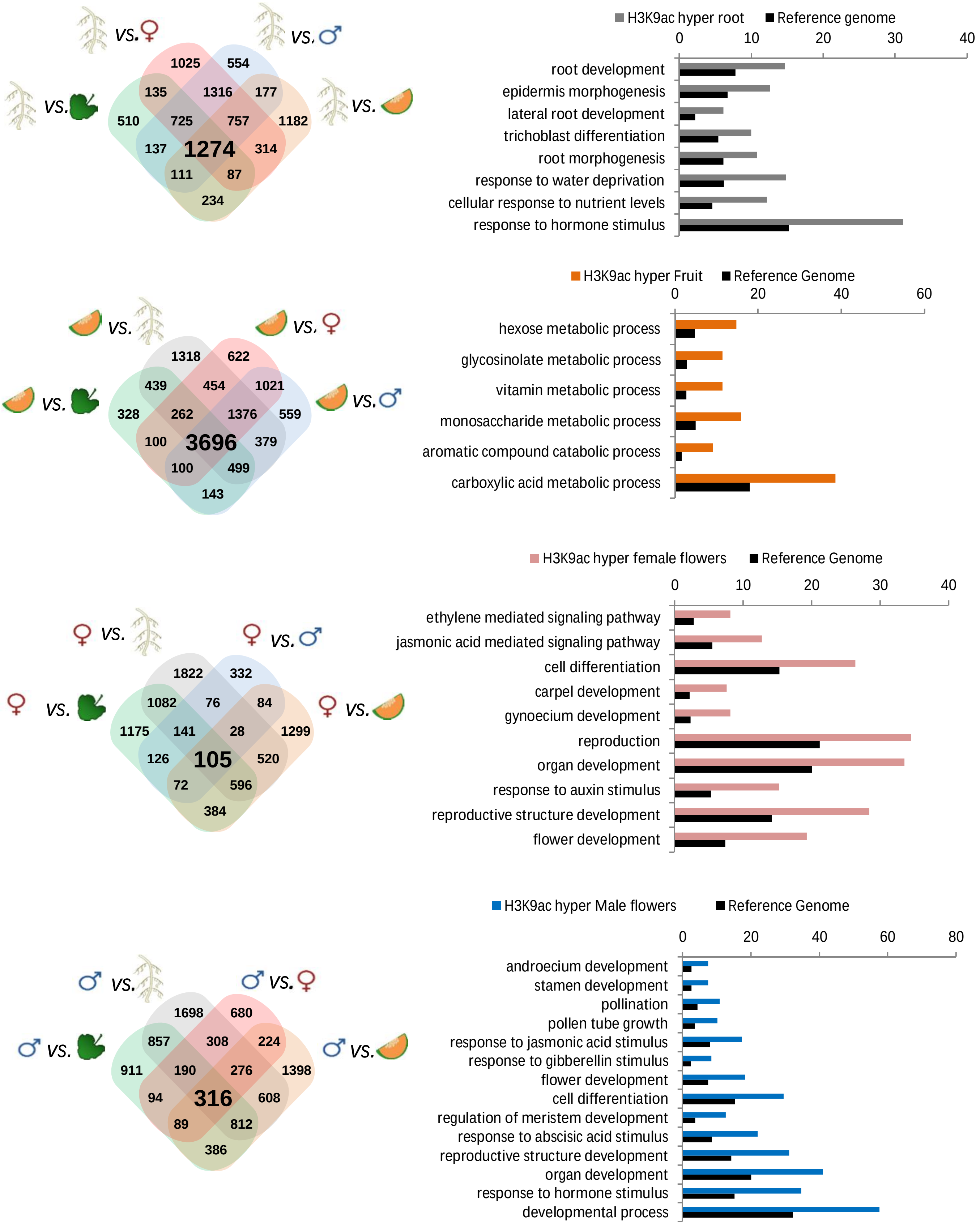

Additional file 1 : Figure S10

H3K9ac hypo-acetylated genes

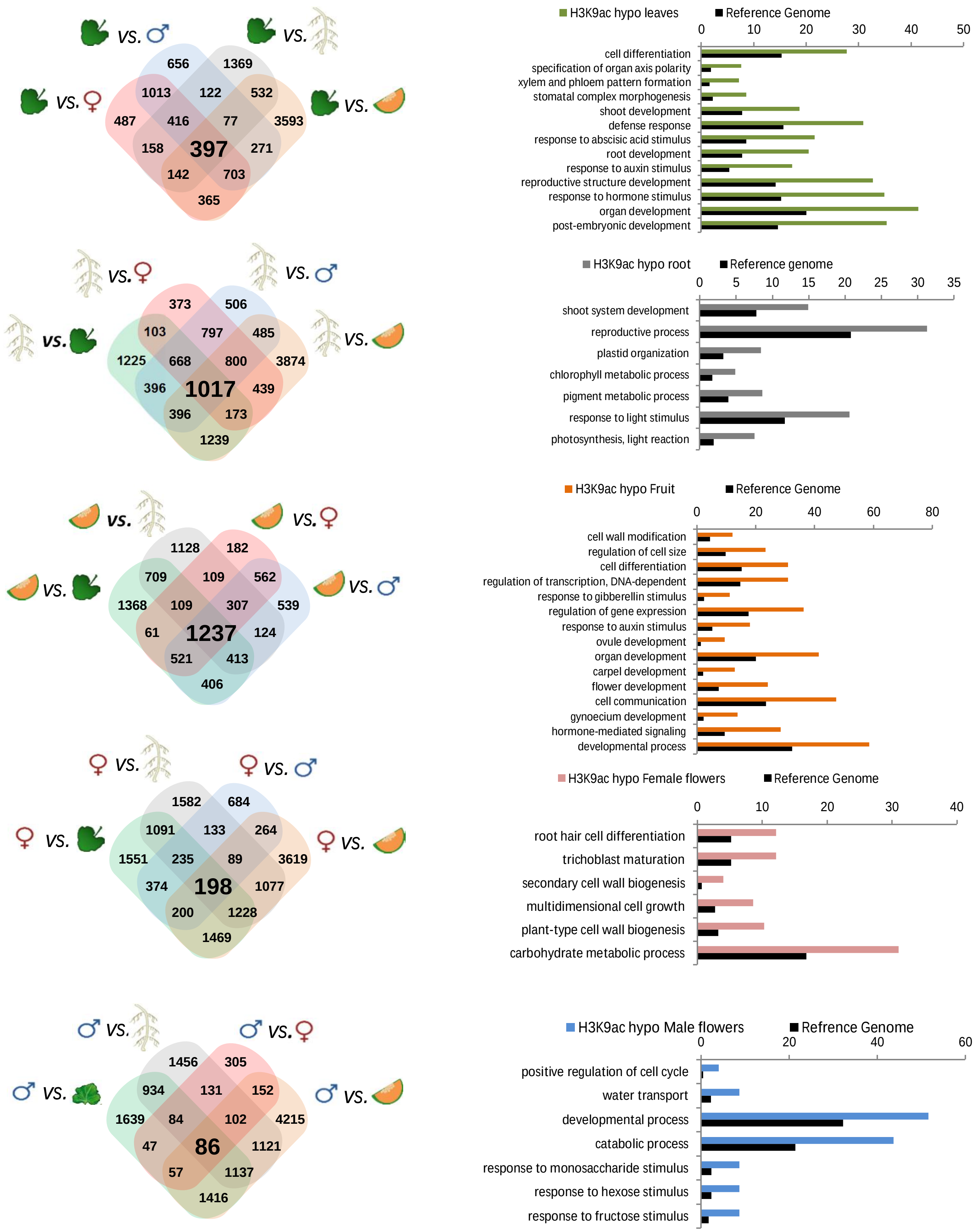

Additional file 1 : Figure S11

H3K27me3 hypo-methylated genes

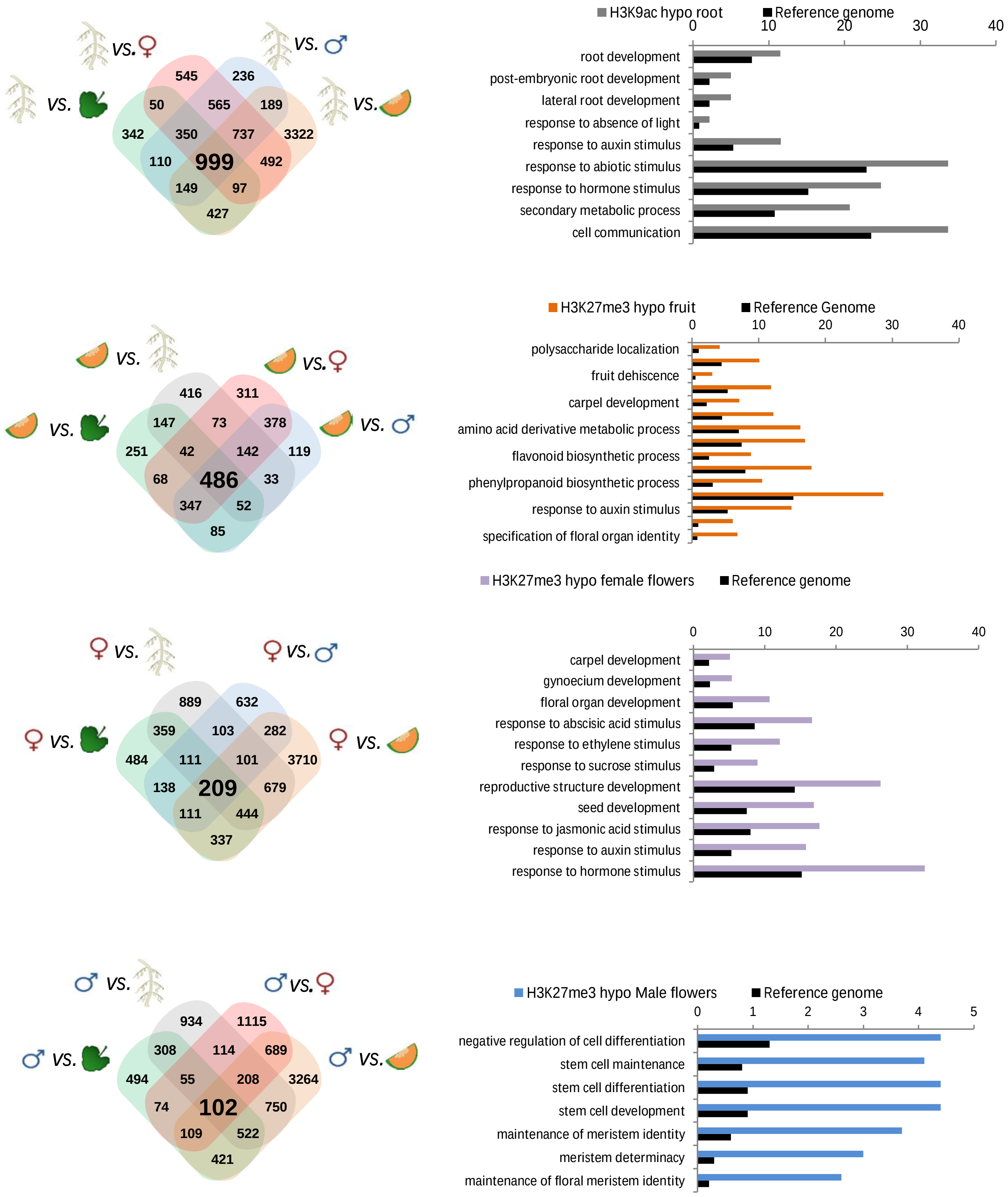

Additional file 1 : Figure S12

H3K27me3 hyper-methylated genes

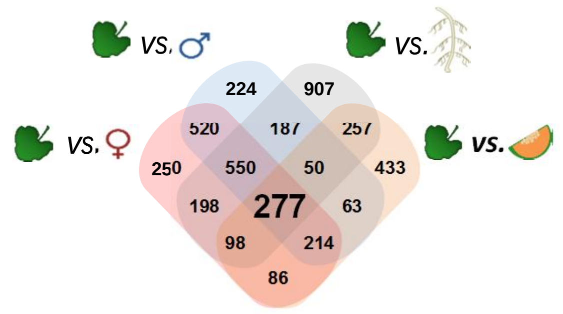

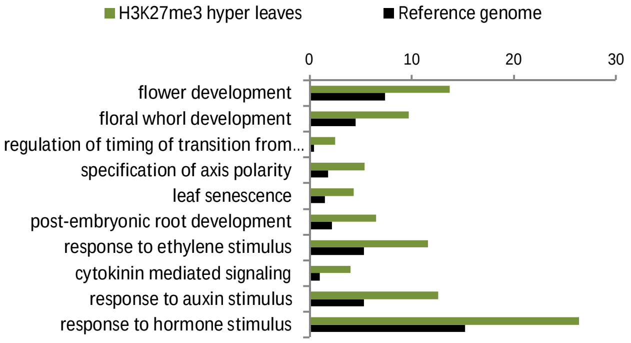

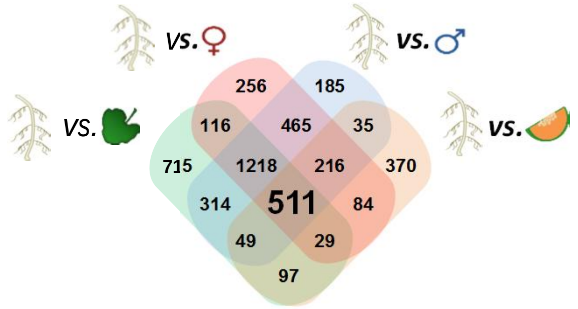

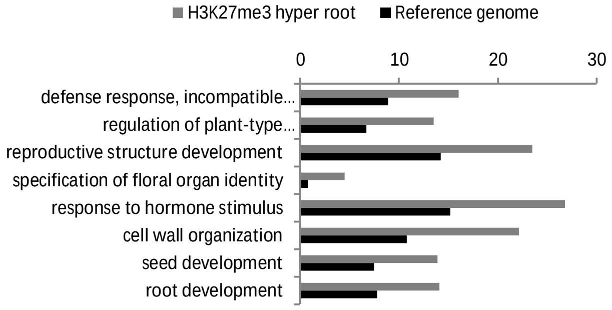

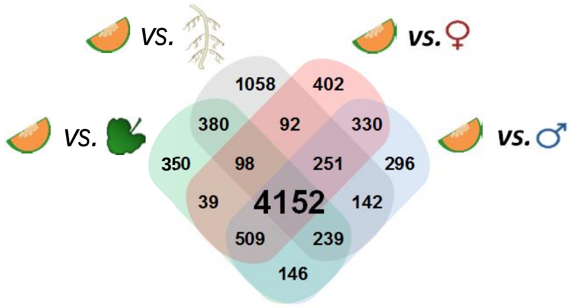

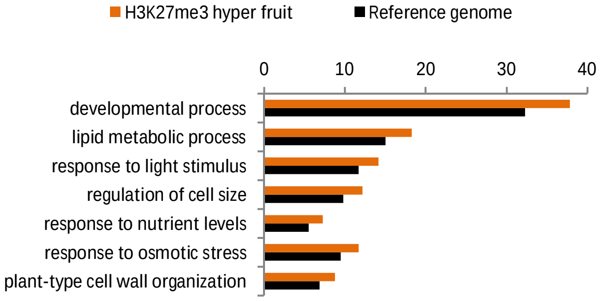

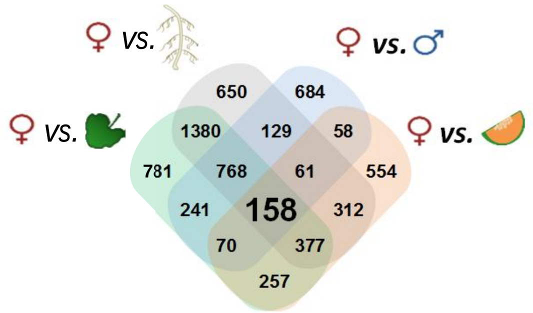

No significant GO process

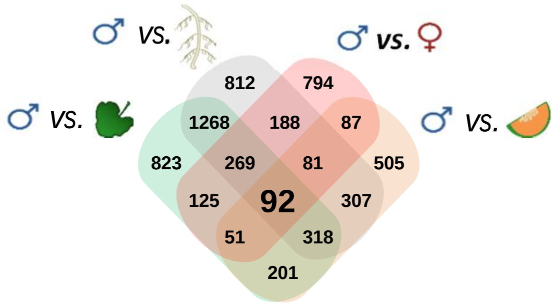

No significant GO process

Additional file 1 : Figure S13

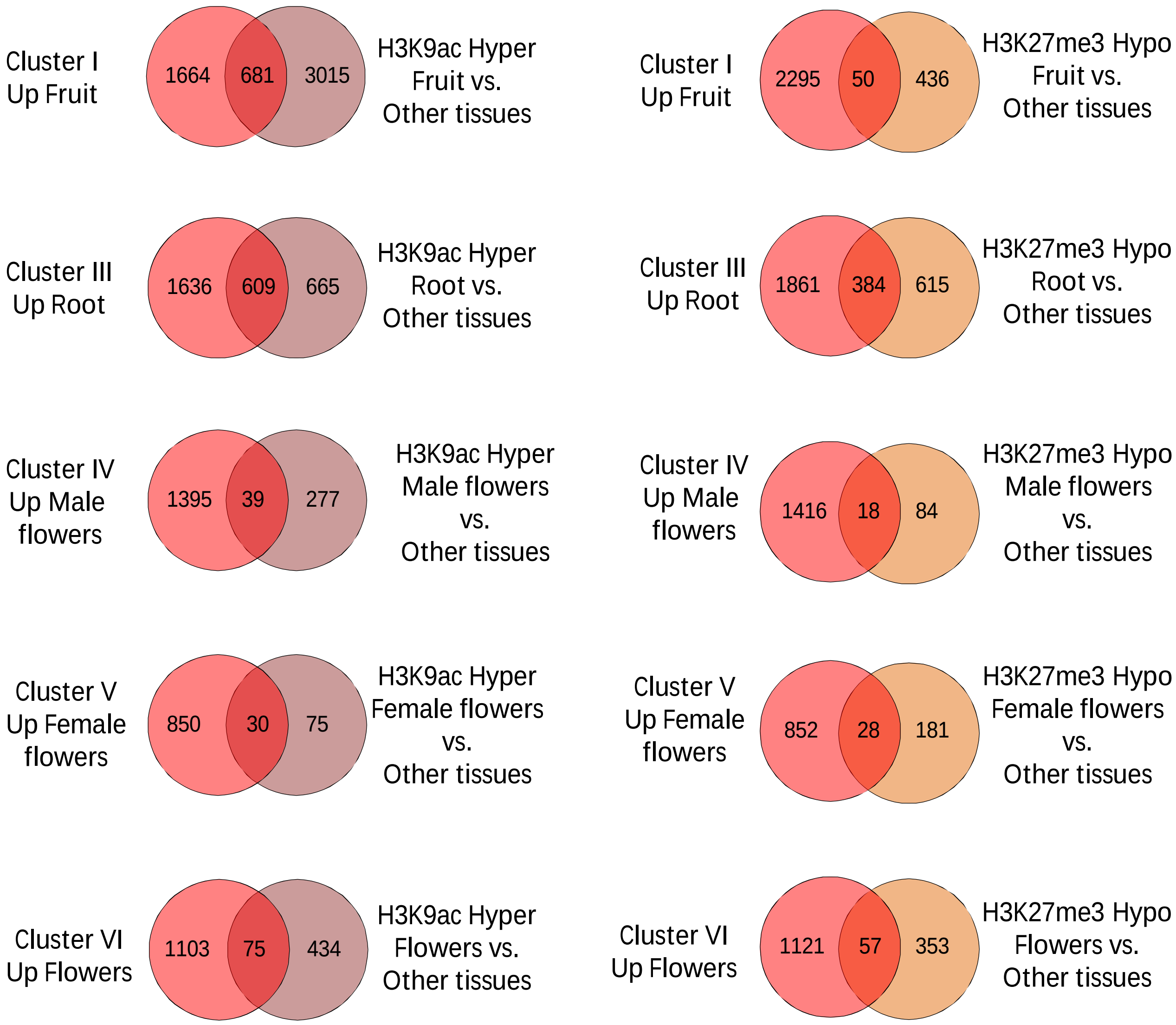

Additional file 1 : Figure S14

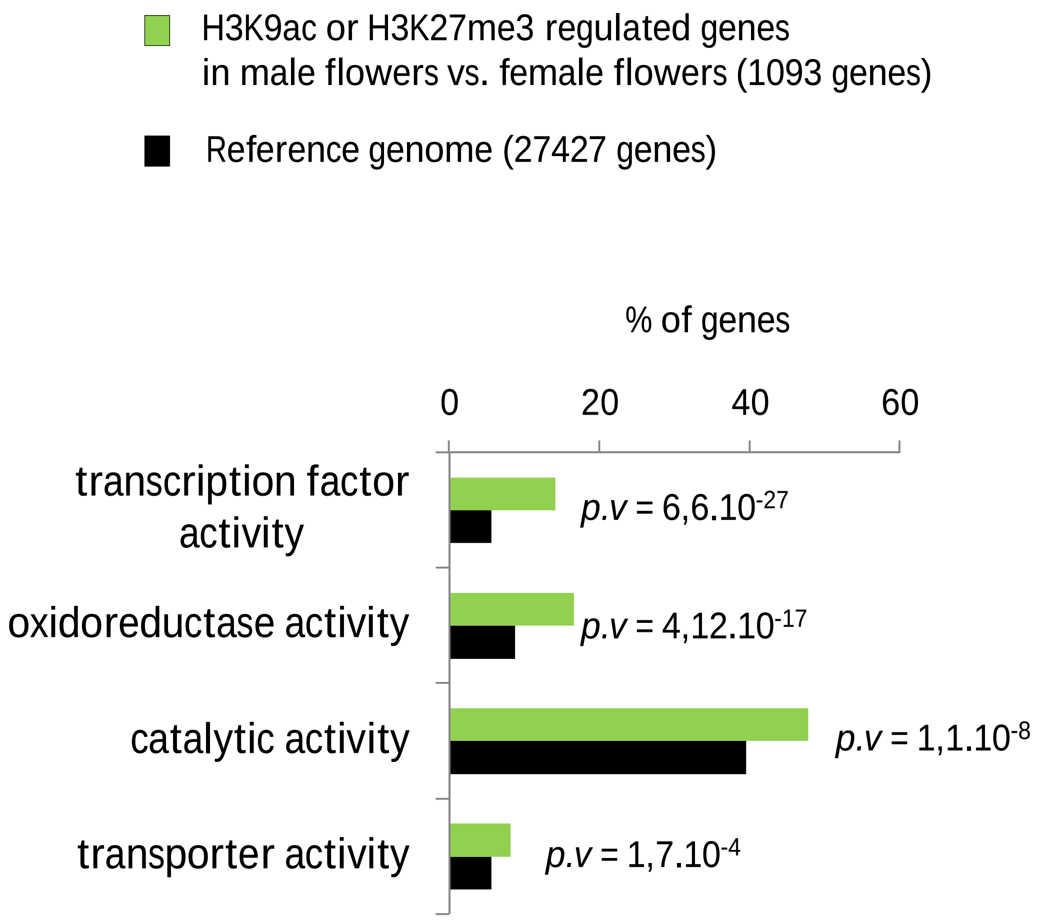

Additional file 1 : Figure S15

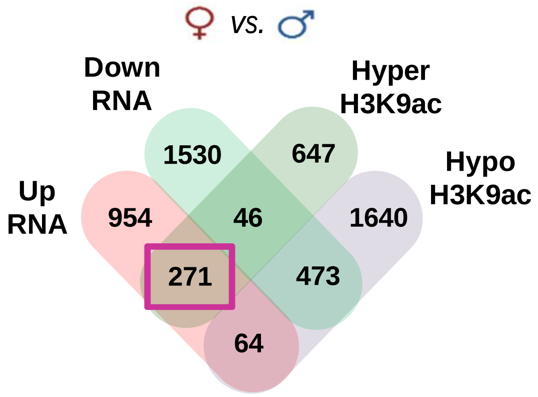

Function

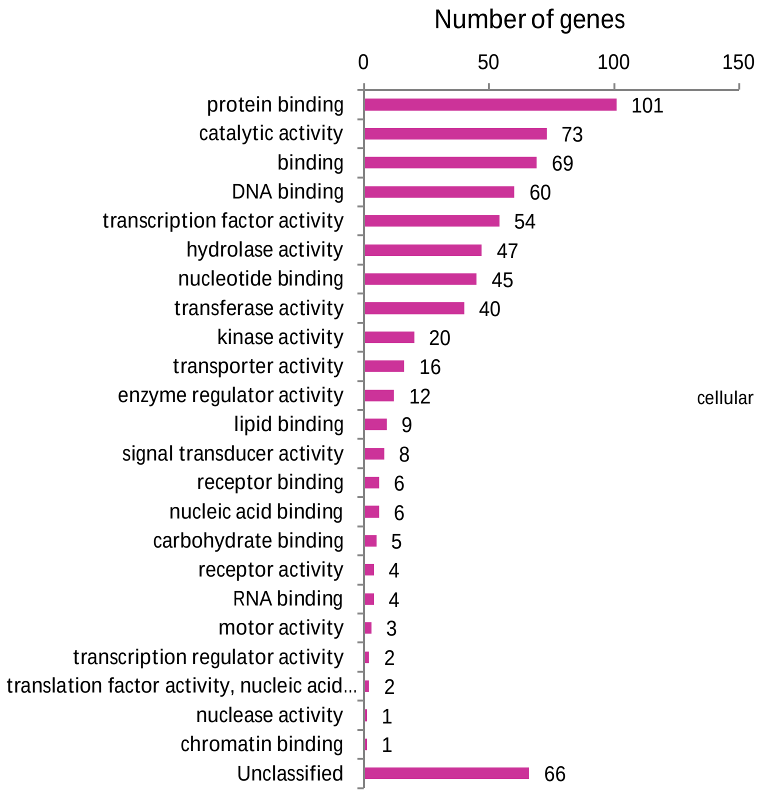

Process

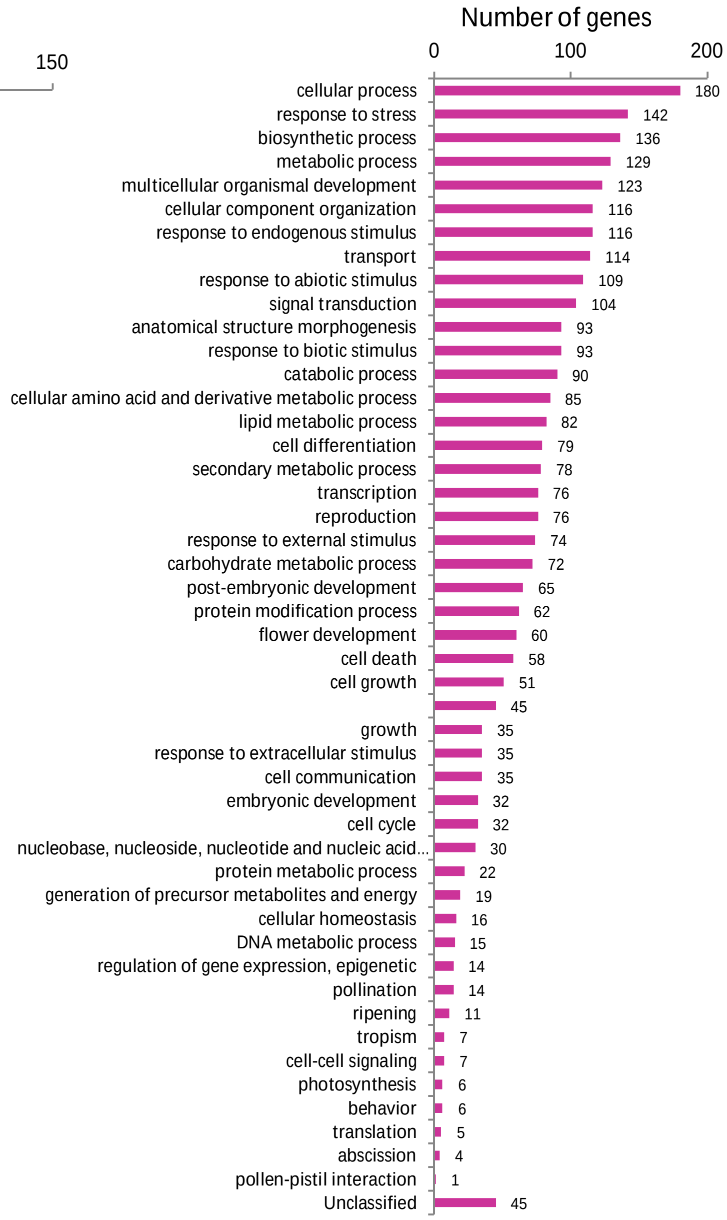

Additional file 1 : Figure S16

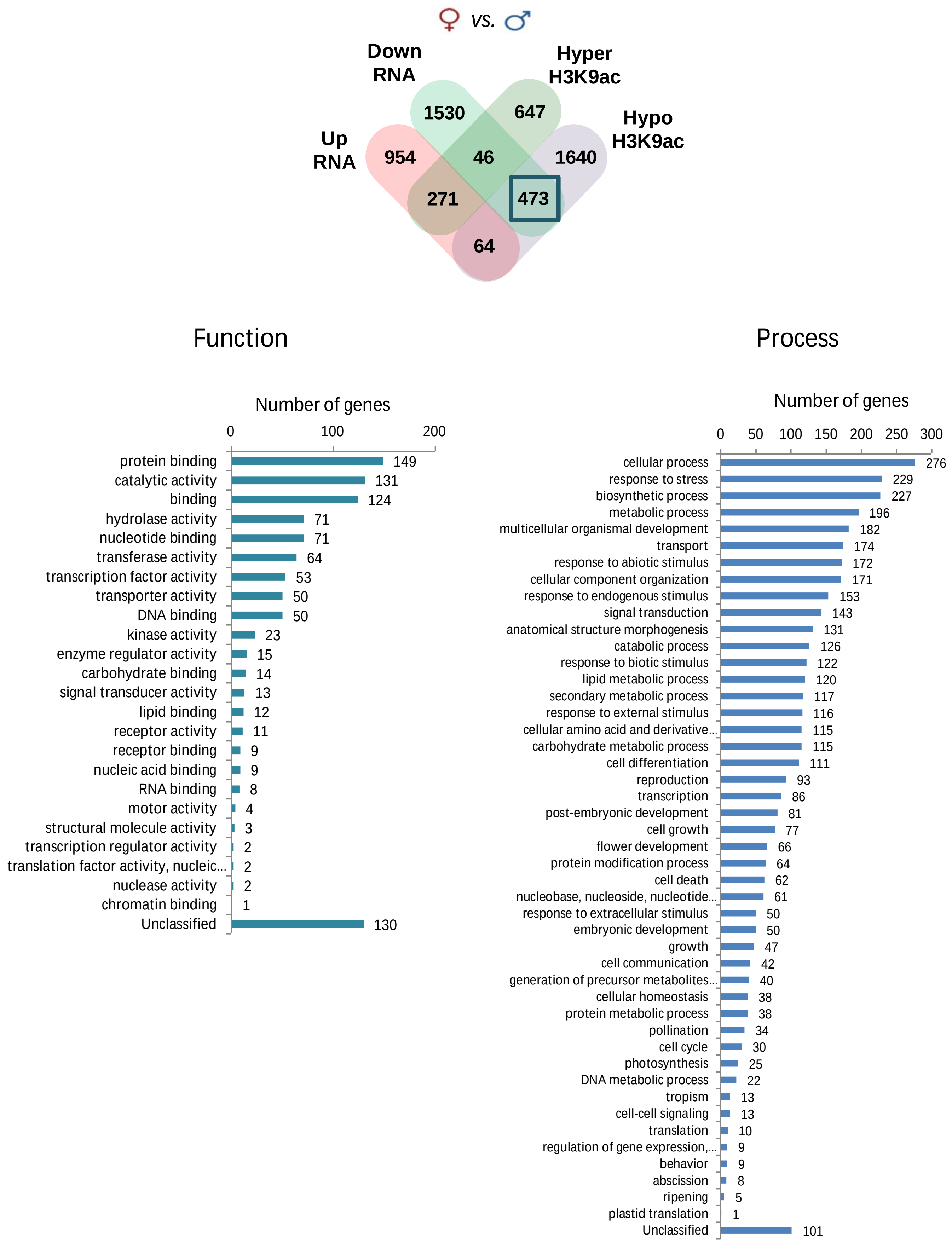

Additional file 1 : Figure S17

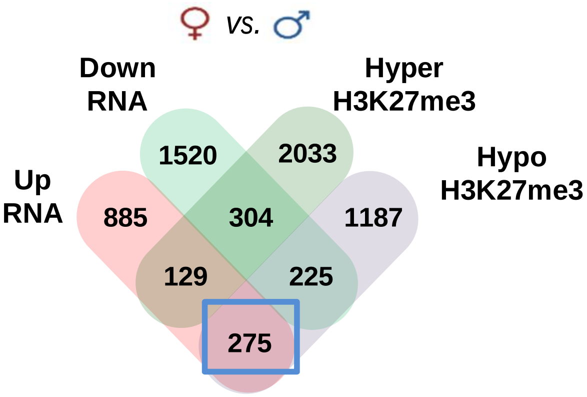

Function

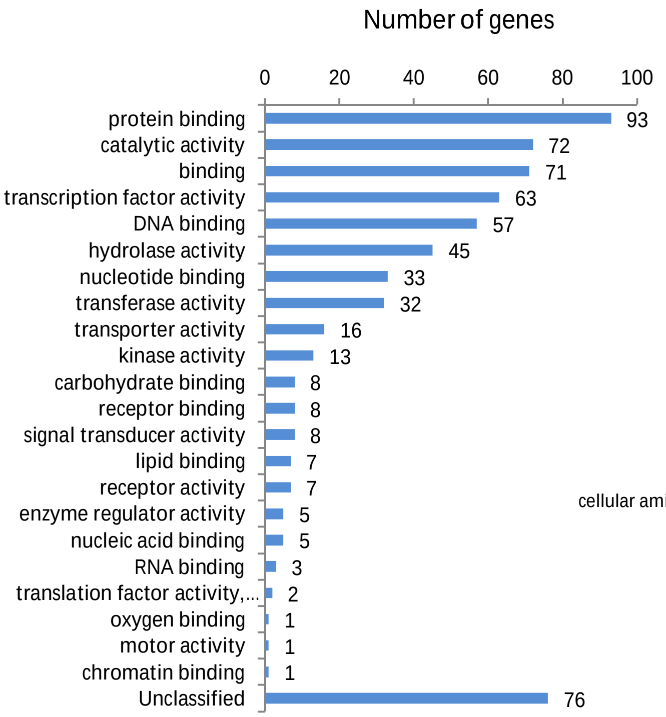

Process

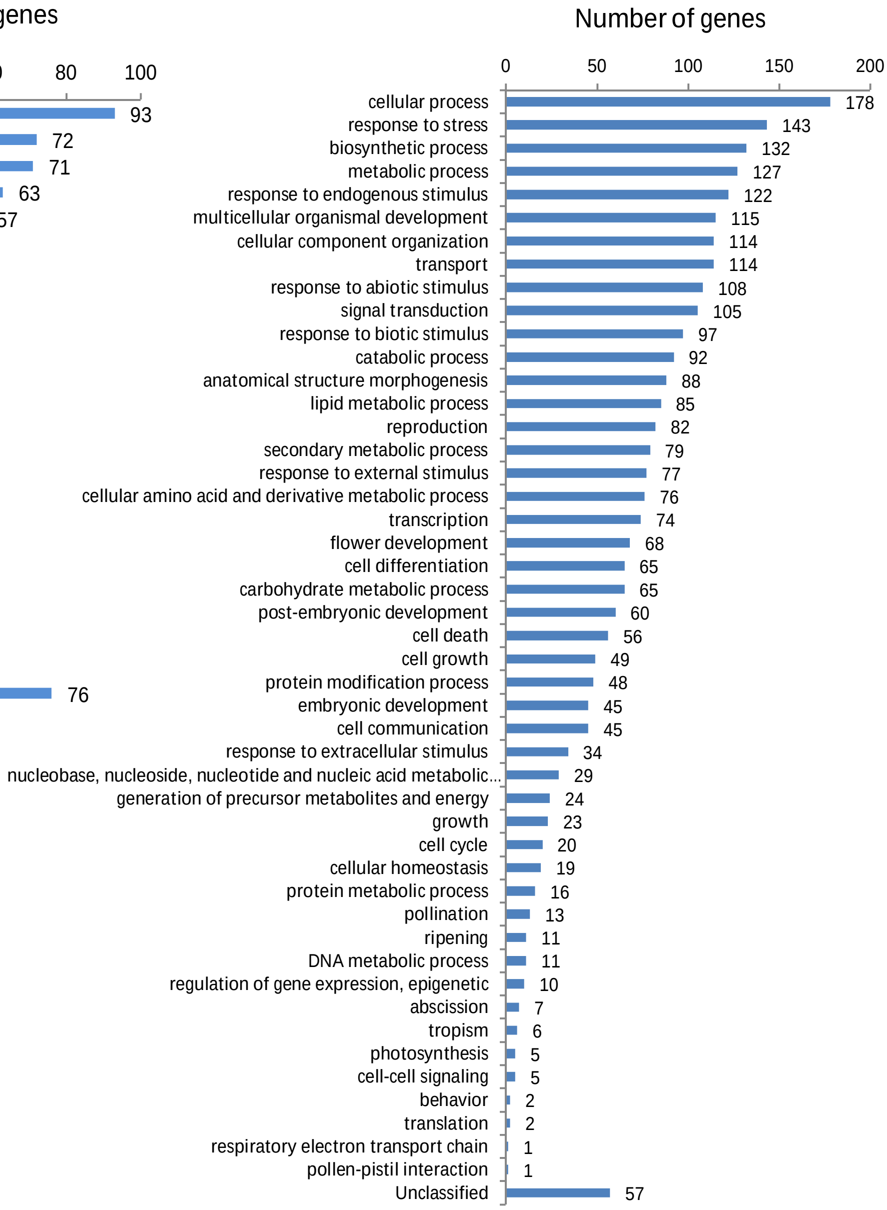

Additional file 1 : Figure S18

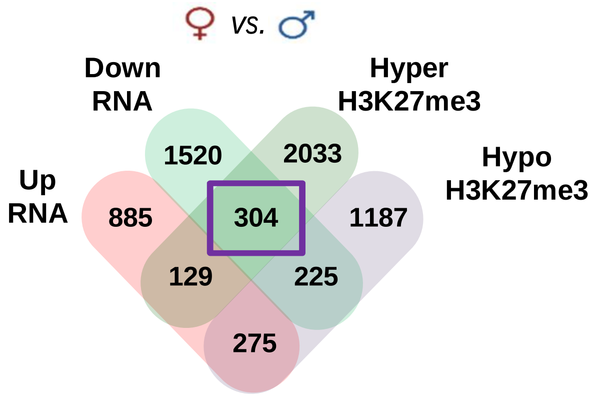

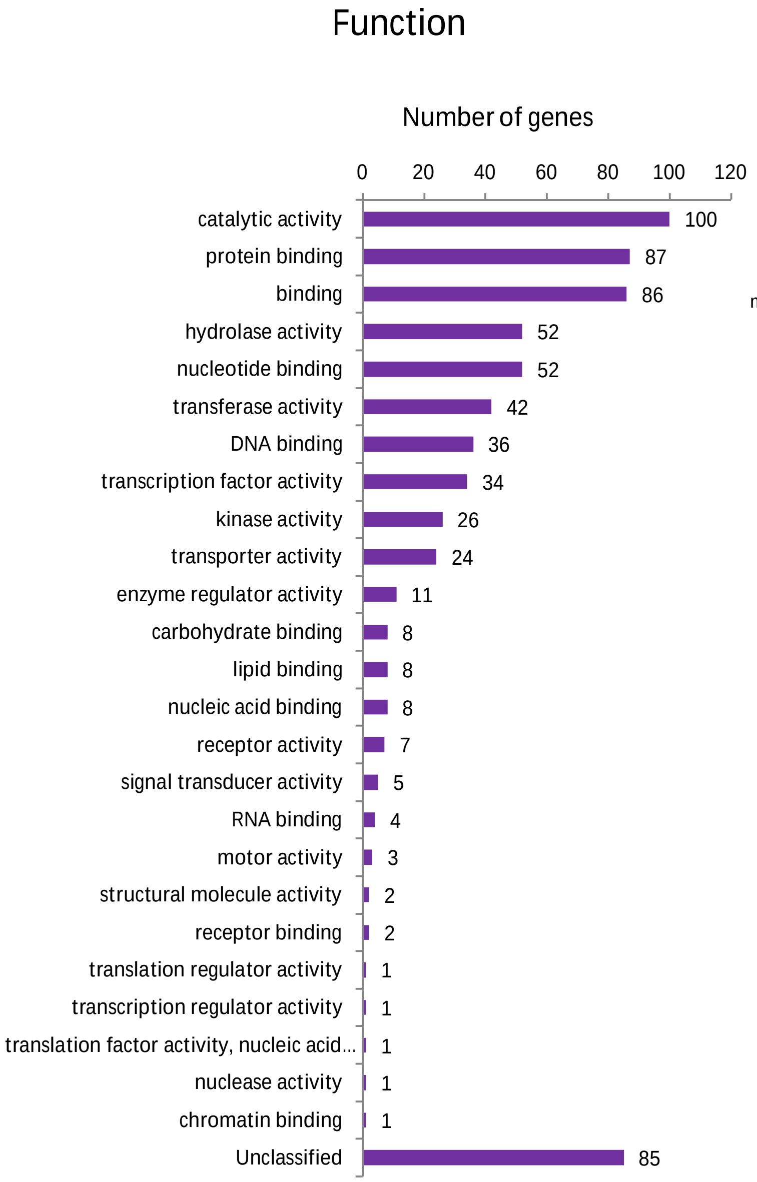

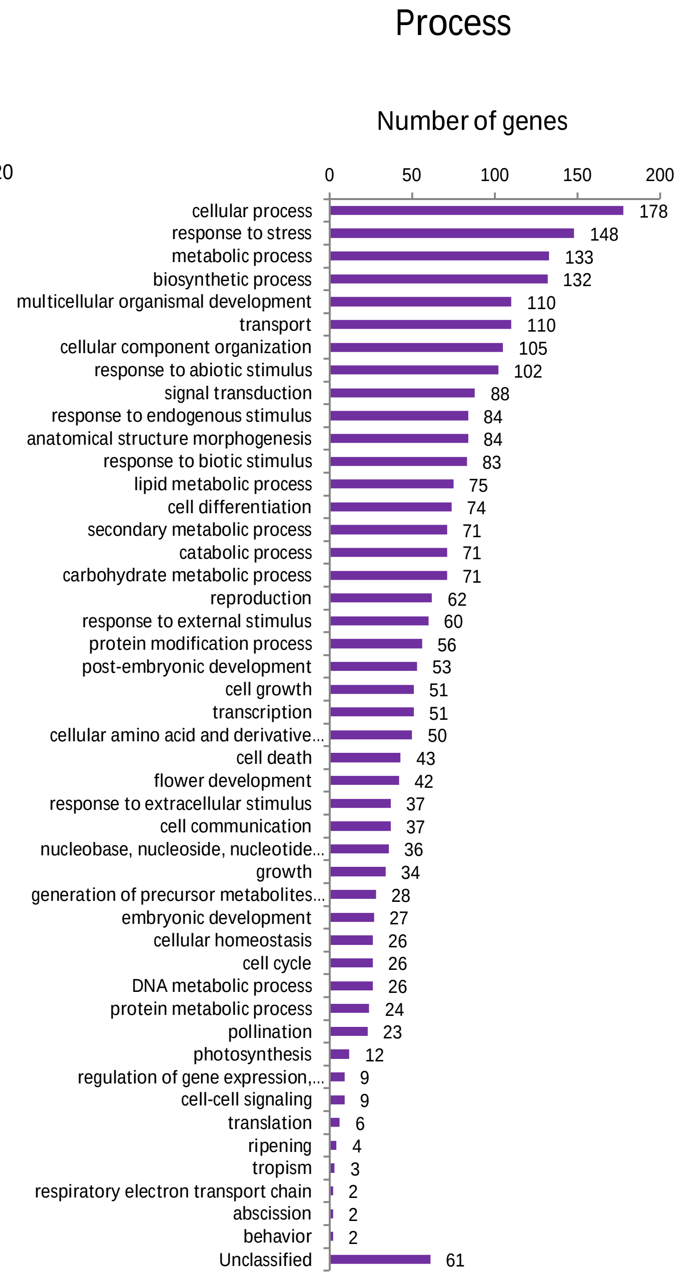

Additional file 1 : Figure S19

MELO3C115188
(Negative control)

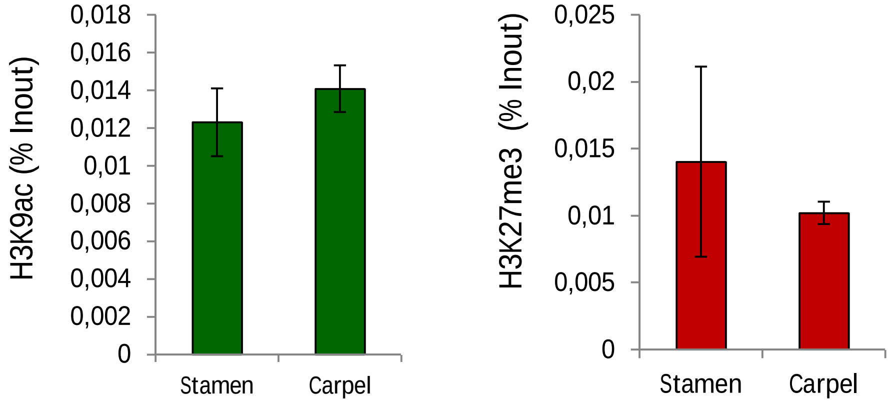

Additional file 1 : Figure S20

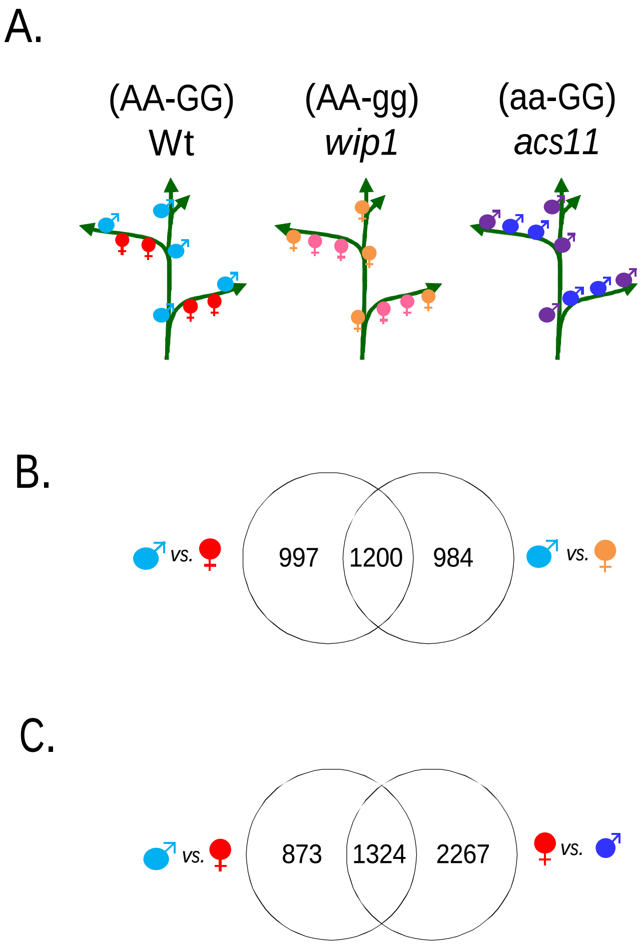

Additional file 1 : Figure S21

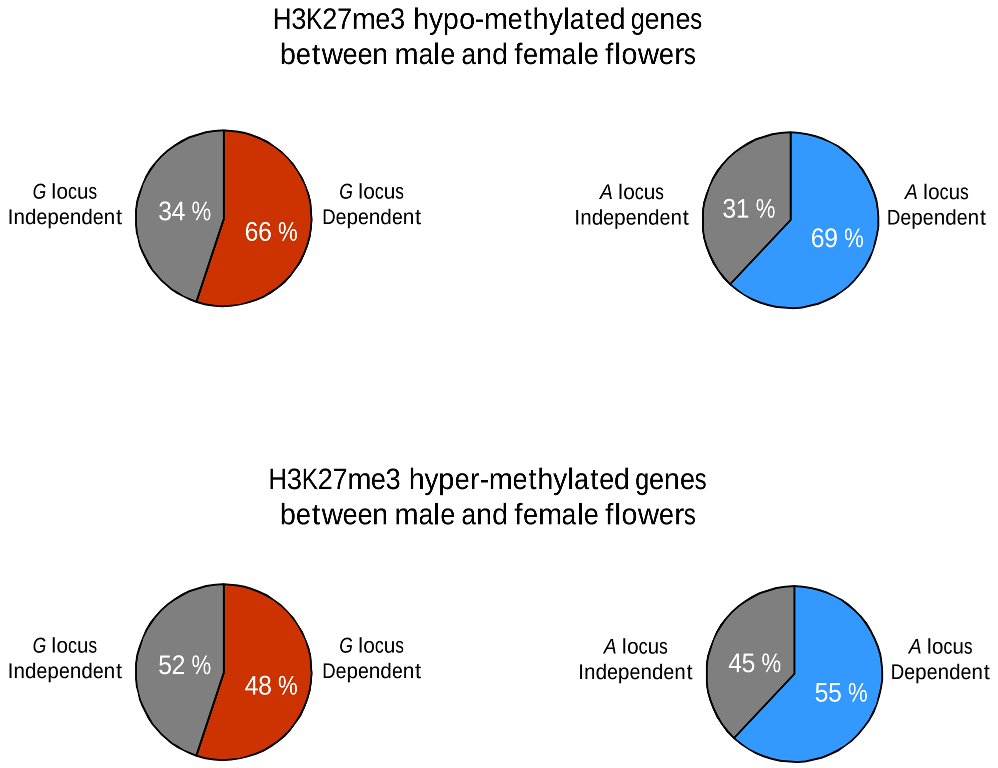

Additional file 1 : Figure S22

H3K27me3 differentially marked genes between male and female flowers

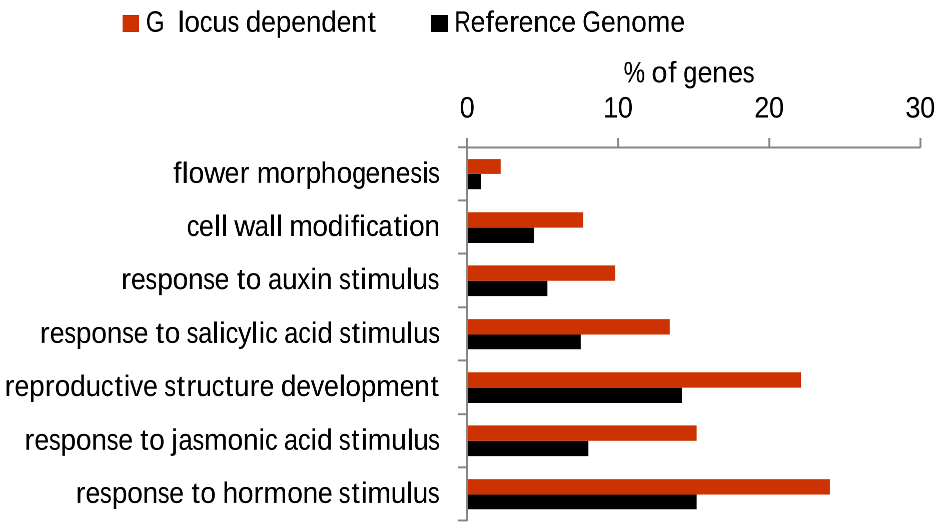

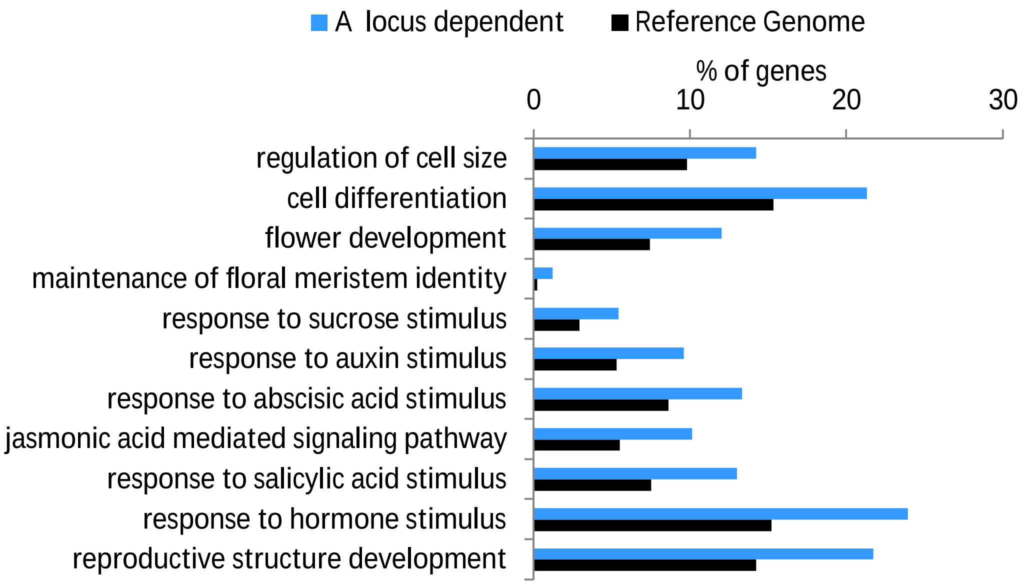

Additional file 1 : Figure S23

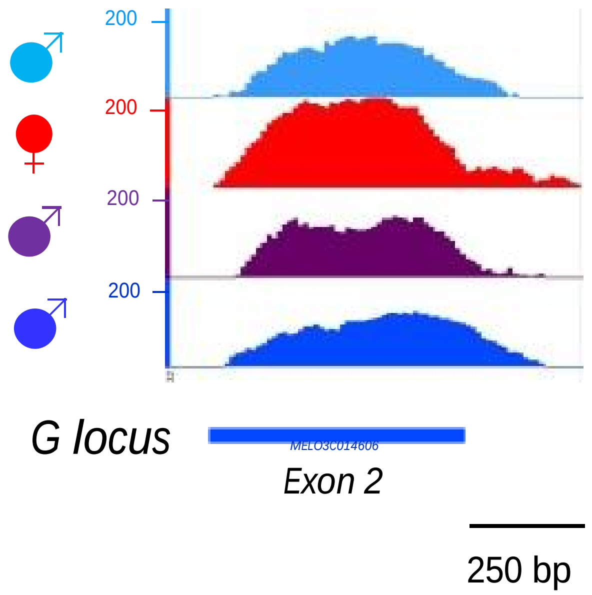

Supplement: Supplementary file 1 — Additional file 1: Figure S1. Pearson correlation between leaves and roots ChIP-seq replicates. The color scale represents the degree of correlation between replicates. Figure S2. Example of melon genomic regions showing ChIP-seq signals of biological replicates in leaves. Genes are represented in blue. Comparisons of peak positions and peak intensities illustrate the high correlation between the two biological replicates. Figure S3. Distribution of mapped reads for H3K27me3 (red shades) and H3K9ac (green shades) along the 12 melon chromosomes of leaves, roots, fruit, male and female flowers. Local peak densities of each epigenetic mark were plotted against the genetic distance (gray) and annotation of sense (dark blue) and antisense transcripts (light blue). Figure S4. Number of H3K9ac and H3K27me3 peaks identified in the different melon tissues. The computational methods MACS2 and SICER were used to determine H3K9ac and H3K27me3 target regions, respectively. Figure S5. H3K27me3 and H3K9ac distribution at the chromosome level in different melon organs. Distribution of H3K9ac (Green), H3K27me3 (red) and annotated genes (gray) are plotted along the chromosome 1. Figure S6. H3K9ac and H3K27me3 peaks distribution on chromosome 3 illustrating the enrichment of the marks in gene-rich regions located at the distal part of the chromosome. Figure S7. Boxplot showing differential peak length between H3K9ac and H3K27me3 in different melon tissues. Figure S8. Average tag density profile of H3K27me3 and H3K9ac along the gene body in different melon tissues. ChIP-Seq densities of equal bins were plotted along the gene body and 2-kb region flanking the TSS or the TES. Figure S9-S12. Tissue-specific H3K9 or H3K27me3 differentially marked genes. Venn diagrams of paired comparisons of each tissue vs. all the tissues are shown on the left. The central overlap corresponds to H3K9 or H3K27me3 specifically modified genes in each tissue. Gene ontology analysis of these subsets of genes i [file 13072_2017_132_MOESM1_ESM.pdf]
